# Immunogenic SARS-CoV-2 Epitopes: In Silico Study Towards Better Understanding of COVID-19 Disease—Paving the Way for Vaccine Development

**DOI:** 10.3390/vaccines8030408

**Published:** 2020-07-23

**Authors:** Vipin Ranga, Erik Niemelä, Mahlet Z. Tamirat, John E. Eriksson, Tomi T. Airenne, Mark S. Johnson

**Affiliations:** 1 Structural Bioinformatics Laboratory, Biochemistry, Faculty of Science and Engineering, Åbo Akademi University, 20520 Turku, Finland; vipin.ranga@abo.fi (V.R.); mahlet.tamirat@abo.fi (M.Z.T.); tomi.airenne@abo.fi (T.T.A.); 2 Cell Biology, Faculty of Science and Engineering, Åbo Akademi University, 20520 Turku, Finland; erik.niemela@abo.fi (E.N.); john.eriksson@abo.fi (J.E.E.); 3 Turku Bioscience Centre, University of Turku and Åbo Akademi University, 20520 Turku, Finland

**Keywords:** SARS-CoV-2, COVID-19, SARS-CoV, in silico analysis, MHC class I epitopes, HLA, viral peptides, antigen presentation, vaccine development, immunoinformatics, homology modeling, molecular dynamics simulations, structural biology

## Abstract

The emergence of the COVID-19 outbreak at the end of 2019, caused by the novel coronavirus SARS-CoV-2, has, to date, led to over 13.6 million infections and nearly 600,000 deaths. Consequently, there is an urgent need to better understand the molecular factors triggering immune defense against the virus and to develop countermeasures to hinder its spread. Using in silico analyses, we showed that human major histocompatibility complex (MHC) class I cell-surface molecules vary in their capacity for binding different SARS-CoV-2-derived epitopes, i.e., short sequences of 8-11 amino acids, and pinpointed five specific SARS-CoV-2 epitopes that are likely to be presented to cytotoxic T-cells and hence activate immune responses. The identified epitopes, each one of nine amino acids, have high sequence similarity to the equivalent epitopes of SARS-CoV virus, which are known to elicit an effective T cell response in vitro. Moreover, we give a structural explanation for the binding of SARS-CoV-2-epitopes to MHC molecules. Our data can help us to better understand the differences in outcomes of COVID-19 patients and may aid the development of vaccines against SARS-CoV-2 and possible future outbreaks of novel coronaviruses.

## 1. Introduction

The ongoing pandemic outbreak of COVID-19 has resulted in the declaration of a global health emergency around the world on 30 January 2020 by the World Health Organization (WHO) [1]. The first reported case was on 31 December 2019 from the Chinese city of Wuhan, from which the virus quickly spread to 213 other countries and territories and, as of 17 July 2020, resulted in at least 13.6 million infections and over 585,000 deaths [2]. Based on the early epidemiology of the COVID-19 disease statistics, the WHO estimated that the fatality rate of the novel coronavirus is around 4%, significantly higher than the mortality rate caused by common human coronaviruses [3]. There are currently numerous scientific, clinical, and socio-economical efforts aimed at combating the COVID-19 disease and its ramifications around the world [4,5].

COVID-19 is caused by Severe Acute Respiratory Syndrome Coronavirus 2 (SARS-CoV-2), which is a positive-sense single-stranded RNA virus belonging to the family Coronaviridae. Coronaviruses usually infect animals, and in humans they generally cause mild respiratory infections with similar symptoms observed in the common cold [6]. However, in the case of the newly recognized SARS-CoV-2 virus, older patients are especially at risk of developing moderate to severe acute respiratory distress syndrome (ARDS), which often requires mechanical ventilation for several weeks [7]. More recently, an increased risk for children for an extremely rare Kawasaki-like disease, acute pediatric vasculitis, has also been observed [8]. In 2003, a coronavirus (SARS-CoV) originating from Southern China caused an epidemic with an estimated case-fatality rate of around 14% [3]. In 2012, in Saudi Arabia, another coronavirus was responsible for Middle East Respiratory Syndrome (MERS), having an even higher estimated case-fatality rate of 35% [9]. However, no new cases of SARS-CoV have been reported since 2004 and MERS-CoV has only caused sporadic outbreaks in a few countries, whereas the recent SARS-CoV-2 has spread around the world, causing a global pandemic [3].

SARS-CoV, MERS-CoV and SARS-CoV-2 all belong to the betacoronavirus genus with a genome size of approximate 30 kilobases that encodes many structural and non-structural proteins, the former coded by their own open reading frames (ORFs) and the latter by two overlapping replicase genes—*ORF 1a* and *1b*—that encode polyproteins 1a (450 kDa) and 1 ab (750 kDa) [10,11]; processing of the polyproteins must take place before the virus can replicate and re-infect other cells and other humans. Two non-homologous, virally encoded cysteine proteinases are involved in processing the polyproteins: the main protease (Mpro), also known as the chymotrypsin-like protease (3CLpro) (see Denesyuk 2020 [12], comparison of the family active sites), and the papain-like protease (PLpro), both of which first autocatalytically release themselves before cleaving out the individual proteins from the polyproteins. The structural proteins include the spike (S) glycoprotein, the envelope (E) protein, the membrane (M) protein and the nucleocapsid (N) protein [10,11].

The coronavirus entry into its host cells is mediated by the S protein, a homotrimeric transmembrane protein, each 180 kDa monomer comprising two functional subunits S1 and S2 [13]. The S1 subunit has two domains, the *N*-terminal domain (NTD) and C-terminal domain (CTD) and, depending on the virus, either NTD or CTD is used as the receptor binding domain (RBD) recognizing host cells. SARS-CoV-2, like most other coronaviruses including SARS-CoV, uses CTD as its RBD for host cell attachment—the RBD of SARS-CoV-2 interacts with the angiotensin-converting enzyme 2 (ACE2) receptor on the host cell [13,14], with about 4X higher affinity in comparison to SARS-CoV [15]. The RBD of bat coronavirus RaTG13 has also been shown to bind human ACE2 and gain cell entry, and the CoV-pangolin/GD RBD shares features consistent with ACE2 binding [15]. The S2 subunit is needed for fusion with the cellular membrane and the fusion is activated by proteolytic cleavage by the human transmembrane serine protease 2 (TMPRSS2), which leads to the internalization of SARS-CoV-2 and replication inside its host cell [13,16]. Thus, infection proceeds but the human host responds by mounting a defense against the onslaught of newly formed virus particles.

The human leukocyte antigen (HLA) system refers to a group of human proteins that are encoded by the major histocompatibility complex (MHC) genes and are critical components of the viral antigen presentation pathway of the immune system [17]. Based on previous reports related to SARS-CoV, there is an increased risk for a severe disease among individuals with the HLA-B*46:01 genotype [18]. Furthermore, epitopes from the S, N and M proteins originating from SARS-CoV have been shown to stimulate immune responses in which CD8+ cytotoxic T cells are vital for eliminating virus-infected cells [19,20,21,22]. Hence, it is of extreme importance to understand this elimination process in order to understand COVID-19 disease progression. The immunological cascade relies on the presentation of viral fragments of infected host cells by the MHC class I cell-surface molecules, which enables recognition by the T-cell receptors (TCR) of the cytotoxic or killer T cells, ultimately leading to the destruction of the infected cells [23]. The viral peptide fragments are created in the cytosol of the host cells mainly by the ubiquitin–proteasome system and the viral fragments are translocated into the lumen of the ER, with the aid of the transporter associated with antigen processing (TAP). In the ER, peptides are loaded on MHC receptors using a peptide loading complex consisting of TAP and several other proteins [23].

In order to narrow down the specific epitopes that could elicit an effective MHC class-I-mediated T cell response, we predicted linear 9-mer immunogenic SARS-CoV-2 peptides and their prominent interacting HLA allotypes using the Immune Epitope Database and Analysis Resource (IEDB) and NetCTL1.2 web servers. The identified peptides were then analyzed in conjunction with the available experimental data for SARS-CoV-derived linear T-cell epitopes. The three-dimensional structural models of selected ternary complexes of SARS-CoV-2 epitope—HLA allotype—T cell receptor were created to assess interactions at the structural level. Our results can at least partially explain individual differences in the COVID-19 severity and could potentially be used for vaccine development against SARS-CoV-2.

## 2. Materials and Methods

### 2.1. Source of Sequences

All 26 protein sequences encoded by the most up-to-date SARS-CoV-2 genomic sequence (RefSeq: NC_045512.2) were retrieved from NCBI RefSeq database [24] on 27 March 2020 (full accession identifiers in Table 1).

### 2.2. MHC Class I Epitope Prediction

The IEDB (http://tools.iedb.org/mhci/) [25] and NetCTL1.2 (http://www.cbs.dtu.dk/services/NetCTL/) [26] web servers were used with default parameters to predict SARS-CoV-2 epitopes and their binding affinities (expressed as IC_50_) to different HLA allotypes. The IEDB server sorts the predicted MHC-I-binding viral epitopes based on the percentile score, which is calculated by comparing the predicted binding affinities of SARS-CoV-2 peptides and affinities calculated for a large set of peptides, randomly selected from the SWISSPROT database [25]; the IEDB server integrates an artificial neural network (ANN), stabilized matrix method (SMM) and Combinatorial library (CombLib). The IEDB method is highly accurate in classifying MHC class I epitopes, having an AUC for the ROC curve greater than 0.9 [27]. The NetCTL1.2 server integrates prediction of proteasomal cleavage, TAP transport and peptide-binding to 12 MHC-I supertypes (see Table S1A for a list of HLA-A and HLA-B allotypes that belong to these 12 supertypes, with data extracted from the published scientific literature [28,29]). The NetCTL1.2 method allows for the identification of class I MHC epitopes with a sensitivity of 0.80 and specificity of 0.97 based on the default filtering threshold score of 0.75 [26]. Since the NetCTL1.2 and IEDB servers use different multistep approaches to predict the binding of SARS-CoV-2 peptides to HLAs, we used both servers.

MHC-I allotypes are known to bind epitopes with lengths of 8 to 11 amino acids. The optimal epitope length was determined by potting the IC_50_ values of the predicted (IEDB) top 1 percentile epitopes against 8- to 11-mers. Moreover, the immunogenicity of the top 1 percentile epitopes were predicted using the MHC-I immunogenicity server of IEDB (http://tools.iedb.org/immunogenicity/) [25]. The epitopes with an immunogenicity score greater than 0.25 were considered for comparison with epitopes predicted using the NetCTL1.2 server.

### 2.3. Comparison of Predicted and Experimentally Known Epitopes

In order to identify experimental epitopes matching the predicted SARS-CoV-2 epitopes, the data of experimentally known SARS-CoV epitopes and their interacting MHC allotypes (validated using T-cell assays and MHC ligand assays) were downloaded from the IEDB database on 15 April 2020 (https://www.iedb.org/database_export_v3.php) [25]. The characteristics of each “match” (protein name, sequence, mapped start-end, MHC-I allotype, etc.) were tabulated and are presented in Table S8.

### 2.4. Epitopes Physicochemical Properties and eMHC-I Complex Stability

The grand average of hydropathicity index (GRAVY) was calculated using the Kyte–Doolittle hydropathy index scale [30]. TMHMM2.0 was used to search potential transmembrane helices [31]. The Jpred4 web server was used to predict secondary structures in non-transmembrane proteins [32]. The half-lives of the predicted eMHC-I complexes was predicted using the NetMHCstabpan1.0 web server [33]. Data were plotted using GraphPad Prism (GraphPad software, version 8.0.0; https://www.graphpad.com).

### 2.5. Docking and Structural Analyses

The crystal structures of an influenza A virus epitope in complex with the HLA-A*02:01 allotype (PDB ID: 5TEZ, chain A and C; 1.7 Å) [34] and hepatitis B core antigen with HLA-A*02:06 (PDB ID: 3OXR, chain A and C; 1.7 Å) [35] were downloaded from the Protein Data Bank (PDB) [36] (see Table S1B for the PDB codes of MHC-I allotype structures indicated in Table S1A). For docking to HLA allotypes, the Rosetta FlexPepDock web server [37] was used, after the 9-mer influenza A epitope was mutated to match selected SARS-CoV-2 epitopes using PyMOL (The PyMOL Molecular Graphics System, Schrödinger, LLC). For ternary complex analysis, the T-cell receptor (TCR)-HLA-influenza A epitope complex (PDB ID:5TEZ) was used as a template. Coordinates of the TCR α and β chains (PDB ID: 5TEZ; chain I and J, respectively) and the docked epitope–HLA-A*02:01 complex were saved using PyMOL, and interacting residues visually inspected at the interface.

### 2.6. Molecular Dynamics Simulations

Molecular dynamics (MD) simulations followed the general protocol detailed in [38]. Briefly, the ternary composite structure of HLA-A*02:01 in complex with the SARS-CoV-2 S protein epitope ^1220^FIAGLIAIV^1228^ and TCR was used as a starting structure to perform molecular dynamics simulation. The structure was initially prepared using Maestro (Schrödinger Release 2019–1: Maestro, version, Schrödinger, LLC) by adding hydrogen atoms, determining the protonation states of ionizable side-chains and energy minimizing the structure to remove bad contacts. Three independent simulations were carried out using the Amber program (version 2018) [39] and the ff14SB protein forcefield [40]. The ternary structure was solvated with TIP3P water molecules [41] in an octahedral box, keeping a 12 Å distance between solute atoms and the surface of the box. The simulation system was neutralized by adding Na^+^ ions, with additional Na^+^/Cl^−^ ions incorporated to bring the system salt concentration to 0.15 M. The system was then energy-minimized for 5000 cycles using the steepest descent and conjugate gradient methods. The minimization was carried out in six-stages, where a restraint on solute atoms was gradually lowered from 25 to 0 kcal mol^−1^ Å^−2^. Subsequently, the system was heated to 300 K during 100 ps with a 10 kcal mol^−1^ Å^−2^ restraint on solute atoms. Next, equilibration was performed for 6 ns in four steps by systematically reducing the restraint force to 0 kcal mol^−1^ Å^−2^. Finally, a restraint-free 100 ns production simulation was carried out at constant temperature (300 K) and pressure (1 bar). Coordinates were saved every 20 ps and the sampled conformations were analyzed using VMD [42], Cpptraj [43] and Chimera [44] programs. Root–mean–square fluctuation (RMSF) calculation was computed using the Cα atoms of the initial structure as a reference. Hydrogen bonds were defined with a bond length ≤3.5 Å and a bond angle ≥135°.

## 3. Results

### 3.1. Prediction of Binding of SARS-CoV-2-Derived Peptides to MHC Class I Receptors

In order to estimate the potential antiviral cytotoxic T-cell response linked to specific HLA allotypes, we predicted the binding affinity of all possible linear 8- to 11-mer peptides derived from the 26 proteins (Table 1) of the SARS-CoV-2 proteome (N_8_ = 375, N_9_ = 2105, N_10_ = 1556 and N_11_ = 2377) to HLA-A and HLA-B supertypes using the IEDB web server [25]. The HLA-C supertype—an extremely good ligand for killer-cell immunoglobulin-like receptor (KIR) on natural killer (NK) cells—was not selected for this analysis because it is known to be less effective in presenting antigens to cytotoxic T-cells than either HLA-A or HLA-B [45]. The class I MHC-epitope (eMHC-I) complexes were classified into three different groups based on the predicted epitope-to-MHC binding affinity scores [46]: strong binders (IC_50_ ≤ 50 nM), weak binders (50 nM < IC_50_ ≤ 500 nM) and non-binders (IC_50_ > 500 nM).

**Figure 1:**
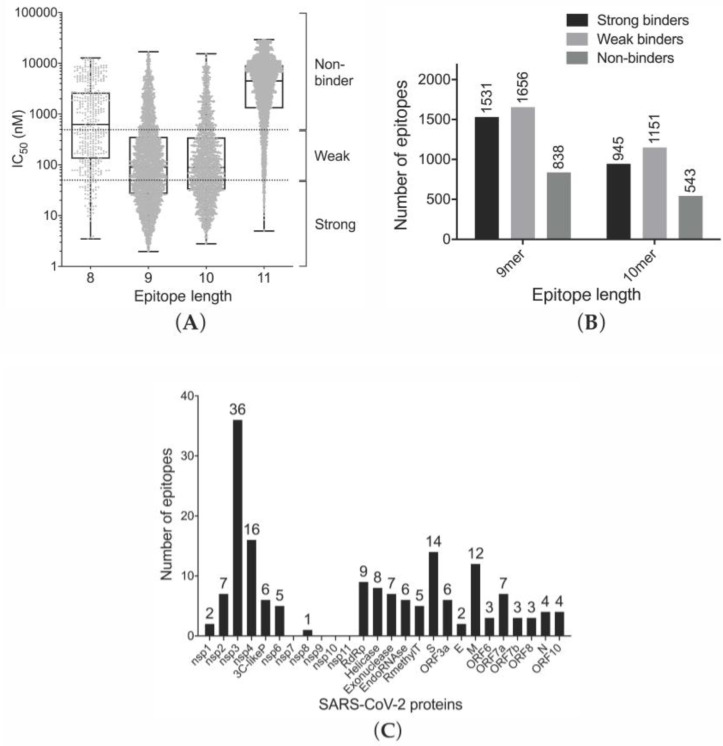
Prediction of binding of antiviral peptides to MHC-I allotypes. (**A**) Distributions of the predicted affinities (IC_50_, log scale) to HLA-A and HLA-B supertypes of all possible linear 8- to 11-mer peptides derived from the 26 proteins of the SARS-CoV-2 proteome. The predicted MHC class I binding epitopes were classified as strong MHC binders (IC_50_ ≤ 50 nM), weak binders (50 nM < IC_50_ ≤ 500 nM) and non-binders (IC_50_ > 500 nM); (**B**) Number of MHC-I–binding 9- and 10-mers categorized as strong, weak and non-binders; (**C**) Number of 9-mer epitopes with an immunogenicity score ≥ 0.25 in SARS-CoV-2 proteins identified with the IEDB and NetCTL1.2 prediction methods.

Out of the highest scoring SARS-CoV-2 epitopes (top 1 percentile), the 9- and 10-mers had, on average, a higher binding affinity to MHC class I supertypes than either the 8- or 11-mer epitopes (Figure 1A). Moreover, there were 52% more 9-mer peptides (3187) predicted to bind to class I MHC receptors (IC_50_ ≤ 500 nM) in comparison with 10-mer peptides (2096) (Figure 1B). Consequently, the top one percentile 9-mer peptides were selected for further analysis.

The predicted (IEDB) SARS-CoV-2 9-mer epitopes (top one percentile) for 50 different HLA allotypes (Table S1A) with an immunogenicity score ≥ 0.25 were compared to those predicted using the NetCTL1.2 server [26]. The MHC class I epitopes identified from this consensus/combined approach were classified using the binding affinity values from IEDB prediction method as strong MHC binders (Table S2), weak binders (Table S3) or non-binders (Table S4). Based on this analysis, many peptides derived from nonstructural proteins (nsp), surface glycoproteins (S) and membrane proteins (M) of SARS-CoV-2 are likely to be presented by MHC class I receptors (Figure 1C, Table S5), and hence have a high potential to activate an immune response or the destruction of infected host cells, with many epitopes being derived from the S, E, 3C-like proteinase (3CLpro) and 3′-to-5′ exonuclease (35EXO) proteins. In order to predict the most potent epitopes, including those without experimental data, we screened in silico the proteins of SARS-CoV-2. We ranked the epitopes based on predicted binding to MHC-I molecules (affinity values; IEDB) and “combined score” (NetCTL1.2), predicting the potency of an epitope to be presented by MHC-I (Tables S6 and S7). The top-ranked, common epitopes from both prediction methods (Table 2) were selected for further analysis; all five of these predicted epitopes are unique to SARS-CoV-2 and not identical in SARS-CoV.

### 3.2. Analysis of Correlation between in Silico Identified SARS-CoV-2 (This Study) and Experimentally Validated SARS-CoV (from IEDB) Epitopes

In order to obtain experimental proof that the MHC-I—Binding SARS-CoV-2 epitopes predicted in this study are presented by the MHC class I antigen processing pathway in vivo, we compared the in-silico-identified 9-mer epitopes (*n* = 166, Table S5) to the equivalent, experimentally identified epitopes of SARS-CoV strains (*n* = 3760; MHC ligand assays data from the IEDB database) [25]. In this comparison, we identified 29 common epitopes of SARS-CoV/SARS-CoV-2 (Table S8) and HLA allotypes HLA-A*02:01 and HLA-A*02:06 molecules as the top antigen presenters; both allotypes having strong binding affinities (IC_50_ ≤ 50 nM) to six peptide epitopes (Table 3): residues ^1220^FIAGLIAIV^1228^ from the S protein, ^17^VLLFLAFVV^25^ and ^20^FLAFVVFLL^28^ to the E protein, ^204^VLAWLYAAV^212^ to 3CLpro, ^330^LLSAGIFGA^338^ to nsp3 and ^184^VLWAHGFEL^192^ to 35EXO of SARS-CoV-2.

To examine the evolutionary conservation or ”sequence stability” of the six common epitopes of SARS-CoV/SARS-CoV-2 binding strongly to HLA-A*02:01 and HLA-A*02:06, we performed a blastp (NCBI) search against the non-redundant database of SARS-CoV-2 [47]. Only one epitope—^330^LLSAGIFGA^338^ from nsp3—was found to have heterogeneity in its sequence (Table 3), whereas the other five epitopes were fully conserved, i.e., not yet having changed during the evolution of SARS-CoV-2, suggesting an important role for these peptide sequences for the virus and, at the same time, making these peptides top-candidate antigens for activating the cytotoxic T-cell response against SARS-CoV-2 itself. The five conserved and experimentally proven epitopes we selected for further analyses.

### 3.3. Efficiency of Epitope Presentation to Stimulate an Immune Response

Since MHC class I molecules must retain bound epitopes long enough at the cell surface to successfully induce T-cell-specific immune responses [48], we estimated the half-life in hours for each of the experimentally identified epitope-HLA complexes (Table 3) using the NetMHCstabpan1.0 web server [33]. This analysis revealed that the HLA-A*02:01 and HLA-A*02:06 allotypes have longer predicted half-lives than HLA-A*68:02 when in complex with the immunogenic epitopes (Table 4): all five HLA-A*02:01-epitope complexes and three out of five HLA-A*02:06-epitope complexes had a predicted half-life longer than 4 h, whereas the single HLA-A*68:02-epitope complex had the lowest half-life of less than one hour.

In order to examine the distribution of the HLA-A*02-antigen complex half-lives in general, we analyzed more than 6500 experimentally known complexes of HLA-A*02 supertypes (see Table S1A for a list of allotypes) bound to bacterial- and viral-pathogen-derived epitopes, which were extracted from the IEDB database [25]. This revealed that immunogenic (IC_50_ ≤ 50 nM) HLA-A*02:01-epitope complexes with predicted half-lives of more than three hours were almost double in number in comparison to the HLA-A*02:06-epitope complexes, whereas no immunogenic HLA-A*68:02-epitope complexes were found that would have a similar half-life (Figure S1A). Interestingly, the amino acid sequences of the α chains of HLA-A*02:01 and HLA-A*02:06 are 99.6% identical; the only difference in the sequences—a phenylalanine to tyrosine substitution—is located at the epitope-binding site and is the likely reason for the difference in the epitope binding affinities and half-lives.

Out of the five most stable epitopes (Table 4), three—^1220^FIAGLIAIV^1228^ (S protein), ^17^VLLFLAFVV^25^ (E protein) and ^20^FLAFVVFLL^28^ (E protein)—were derived from transmembrane proteins and were, as expected, more hydrophobic (GRAVY score > 3) than the ^204^VLAWLYAAV^212^ (3CLpro) and ^184^VLWAHGFEL^192^ (35EXO) epitopes originating from intravirion proteins (Table 4). The epitopes derived from the S and E proteins respectively map to transmembrane helical segments ^1214^WYIWLGFIAGLIAIVMVTIMLCC^1236^ and ^12^LIVNSVLLFLAFVVFLLVTLAIL^34^ that, based on analysis using the TMHMM2.0 web server [31], are bitopic in nature, i.e., the predicted transmembrane helices span the lipid bilayer only once. In fact, our predicted epitopes share features, such as being membrane associated, with the well-studied and clinically important epitopes in HIV [49] and tuberculosis [50]. This supports the idea that the transmembrane helical epitopes of SARS-CoV-2 could potentially stimulate cytotoxic T-cell-mediated immune responses.

In order to assess whether hydrophobic residues are enriched in the immunogenic epitopes, we compared the GRAVY score distribution of the immunogenic (*n* = 1678, IC_50_ ≤ 50 nM) and non-immunogenic (*n* = 2228; IC_50_ > 500 nM) HLA-A*02-bound epitopes of bacterial and viral pathogens (retrieved from the IEDB database) (Figure S1B). We found that, for epitopes with a GRAVY score greater than one (having at least seven non-charged residues in a 9-mer epitope), the immunogenic epitopes were more enriched in hydrophobic residues (55%) in comparison to the non-immunogenic epitopes (19%). Thus, our analysis suggests that HLA-A*02 supertype molecules prefer binding to hydrophobic epitopes (IC_50_ ≤ 50 nM), and this agrees with published results [51]. Moreover, we obtained similar results from our in-silico-identified SARS-CoV-2-derived novel epitope—MHC-I complexes (Table 2): the most potent identified epitopes had long half-lives and were derived from either hydrophobic, transmembrane regions of SARS-CoV-2 or from intravirion proteins that were also found to be mutated among SARS-CoV-2 sequences (Table 5).

### 3.4. Structural Properties of the Peptide-HLA-A*02:01-Complexes Defining T Cell Receptor (TCR) Recognition

**Figure 2:**
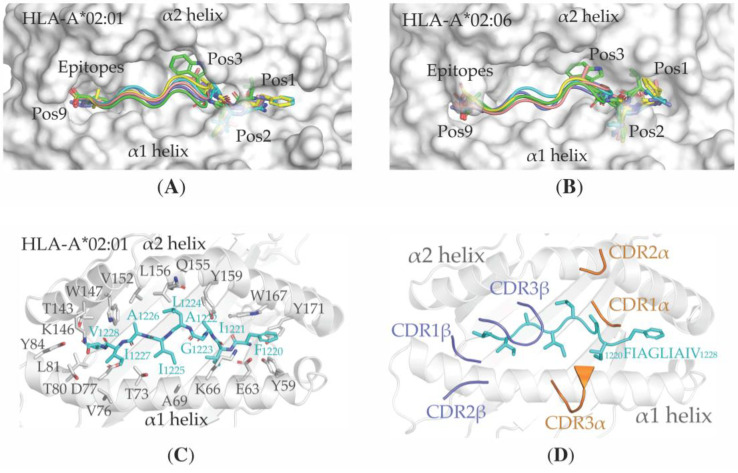

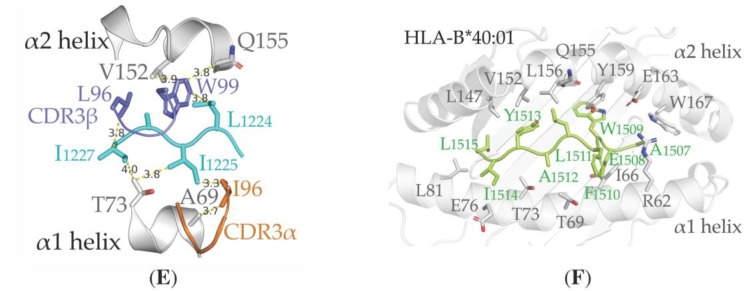
3D structural models of MHC-I in complex with selected epitopes (Table 4) and ternary complex of MHC-I–epitope—T cell receptor (TCR). (**A**) and (**B**) Five immunogenic epitopes (Table 4) were docked into the cleft between the α1 and α2 helices of HLA-A*02:01 (PDB ID: 5TEZ, chain A) and HLA-A*02:06 (PDB ID: 3OXR, chain A) (surface presentation). The epitopes ^1220^FIAGLIAIV^1228^, ^17^VLLFLAFVV^25^, ^20^FLAFVVFLL^28^, ^204^VLAWLYAAV^212^ and ^184^VLWAHGFEL^192^ are respectively colored cyan, yellow, blue, salmon and green, and are shown as ribbons; residues at Pos1-3 and Pos9 as sticks; (**C**) Structure of the epitope ^1220^FIAGLIAIV^1228^ (cyan sticks) docked into the cleft of HLA-A*02:01 (PDB ID: 5TEZ, chain A; gray cartoon and sticks). Residues G1223 to I1227 of the epitope and A69, K66, V76, T80, K146, V152 and Q155 of HLA-A*02:01 have solvent-exposed side chains; (**D**) Ternary complex structure of HLA-A*02:01 (gray cartoon), epitope ^1220^FIAGLIAIV^1228^ (cyan loop and sticks) and TCR. Conformations of the CDR1α, CDR2α and CDR3α loops of the TCR-α chain (orange) and CDR1β, CDR2β and CDR3β of the TCR-β chain (blue) are shown; (**E**) Side chains of residues of CDR3α (orange sticks) and CDR3β (blue sticks) loops making hydrophobic interactions (dotted yellow line; distances in Ångströms) with both the epitope ^1220^FIAGLIAIV^1228^ (cyan sticks) and the HLA-A*02:01 molecule (residues located at the α1 and α2 helices; gray sticks) are shown; (**F**) Structure of the epitope ^1507^AEWFLAYIL^1515^ (green loop and sticks) docked into the cleft of HLA-B*40:01 (PDB ID: 6IEX, chain A; gray cartoon and sticks).

In order to compare the interaction patterns adopted by the predicted top five immunogenic epitopes (Table 4) of MHC-I molecules, we docked the epitopes to the cleft between the α1 and α2 helices of HLA-A*02:01 (PDB ID: 5TEZ, chain A) and HLA-A*02:06 (PDB ID: 3OXR, chain A). This docking analysis agrees with our other prediction data and suggests that the immunogenic epitopes bind to both the HLA-A*02:01 and HLA-A*02:06 allotypes by adopting a similar backbone conformation, as has been observed for the canonical epitope ^1^GILGFVFTL^9^ of the influenza A virus (PDB ID: 5TEZ, chain C) (Figure S1C,D). In more detail, we observed that the residues at position (Pos) 1, 2, 3 and 9 are fully buried within the antigen-binding cleft of HLAs and act as anchoring residues, providing steric constraints to the *N*- and C-terminus of the epitopes (Figure 2A,B). Comparison of the root–mean–square deviation (rmsd) of the superposed Cα atoms of the epitopes revealed a maximum deviation for the ^184^VLWAHGFEL^192^ epitope (35EXO), possibly due to the bulky, aromatic side chain of W186 buried at Pos3 and two charged residues: H188 at Pos5 and E191 at Pos8 (Table 6).

Interactions between the partially solvent-exposed hydrophobic residues at Pos4-Pos8 of the docked epitope ^1220^FIAGLIAIV^1228^ (S protein) and the solvent-exposed residues A69, K66, V76, T80, K146, V152 and Q155 of the α1 and α2 helices of HLA-A*02:01, complement what is seen in the X-ray crystal structure of the influenza A virus epitope-HLA-A*02:01 complex (PDB ID: 5TEZ, chain A and C), and suggest that the SARS-CoV-2 epitope-HLA complex interacts with TCR (Figure 2C).

To understand the molecular basis of TCR binding to the SARS-CoV-2 antigen-loaded MHC-I cell surface molecules, HLA-A*02:01, in complex with the S protein epitope ^1220^FIAGLIAIV^1228^, was superimposed with the HLA-A*02:01 allotype of the influenza A virus ternary complex structure (PDB ID: 5TEZ, chain A), and the atomic coordinates of TCR (TCR-α and TCR-β chain; PDB ID: 5TEZ, chain I and J, respectively) were then utilized for visual analysis of binding. This visualization suggests that the loops CDR2α, CDR1α and CDR3α of the TCR-α chain, and loops CDR2β, CDR1β and CDR3β of the TCR-β chain, recognize the HLA-A*02:01–SARS-CoV-2 ^1220^FIAGLIAIV^1228^ epitope (Figure 2D); the cooperative interacting nature of residues in these loops could provide specificity towards class I MHC molecules. Moreover, the residues L96 and W99 within the CDR3β loop and I96 within the CDR3α loop could be important for antigen–HLA complex recognition due to their likely direct contacts with residues L1224, I1225 and I1227 of the ^1220^FIAGLIAIV^1228^ epitope and residues A69, V152 and Q155 of HLA-A*02:01 (Figure 2E).

**Figure 3:**
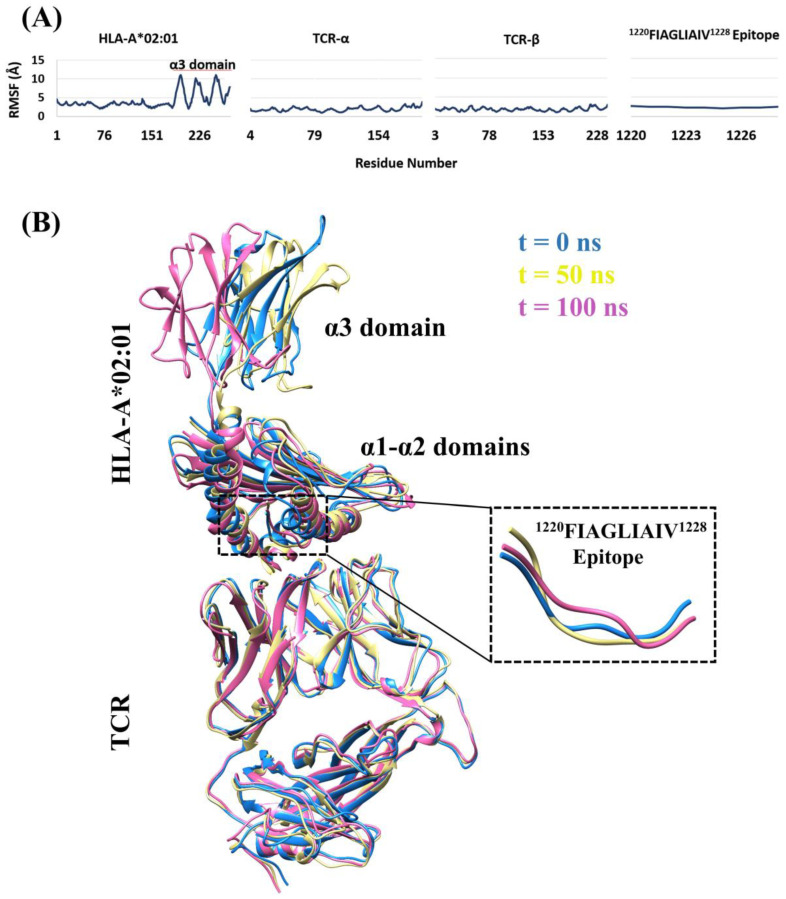
Structural dynamics of the HLA-A*02:01-^1220^FIAGLIAIV^1228^ S protein epitope–T cell receptor (TCR) complex during a 100 ns simulation. (**A**) Cα atom root–mean–square fluctuation (RMSF) of the ternary complex. (**B**) Superimposed conformations of the complex sampled at 0 ns (blue), 50 ns (yellow) and 100 ns (pink) of the simulation. Results from the first of three independent MD simulations are illustrated here.

To assess the conformational and intermolecular interaction dynamics of the HLA-A*02:01-^1220^FIAGLIAIV^1228^ S protein epitope–TCR complex, a 100 ns simulation was carried out on the ternary structure in triplicate. The global conformational dynamics was assessed by computing the RMSF over the Cα atoms (Figure 3A), which shows a stable TCR and epitope, with an average RMSF of 1.86 ± 0.47 Å and 2.36 ± 0.18 Å, respectively. The α1 and α2 domains of HLA-A*02:01 were also stable (3.16 ± 0.48 Å, 3.19 ± 0.46 Å), unlike the α3 domain, which exhibits a higher fluctuation (6.4 ± 2.57 Å) likely arising from the flexibility of the loop between domains α2 and α3 and the lack of stabilizing β2-microglobulin. Figure 3B compares the complex at 0, 50 and 100 ns during the simulation. These observations were consistent among the three independent simulations (Figure S2).

The intermolecular interactions taking place in the complex structure were also examined by visually inspecting the trajectory and calculating the number of hydrogen bonds formed during the simulation (Table S9). For example, in simulation 1, the highest number of hydrogen bond interactions recorded were between HLA-A*02:01 and backbone atoms of the epitope peptide, with T143-V1228 and W147-I1227 interactions topping the list, as they were respectively formed during 98% and 97% of the simulation time. On the other hand, hydrogen bond interactions between the TCR and the epitope were almost exclusively from interactions between W99 (β chain)-I1225 (63%) and Q101 (α chain)-I1225 (10%). The HLA-A*02:01-TCR hydrogen-bonding interactions were mostly between residues from the CDR1α, CDR2α, CDR3α, CDR1β and CDR2β loops of the TCR and the α1 and α2 helices of HLA-A*02:01. Visual analysis also revealed that hydrophobic interactions are integral to the interaction the epitope is making, both with HLA-A*02:01 and the TCR, as the hydrophobic epitope peptide was tightly enclosed by hydrophobic clusters from the proteins throughout the simulation, reflecting observations made on the basis of the original docked complex. Thus, our structural analysis suggests that the S protein epitope ^1220^FIAGLIAIV^1228^ of SARS-CoV-2 (and SARS-CoV), and the other epitopes listed in Table 4, could form strong complexes with HLA-A*02:01 and HLA-A*02:06 allotypes, and that the epitope-HLA complexes can also be recognized by TCRs to initiate cytotoxic T-cell-mediated immune responses.

Similar to the docking results obtained from the experimentally known epitopes with the HLA-A*02 supertype, we observed that residues at Pos1-3 and Pos9 (A1507, E1508, W1509 and L1515) of the novel epitope ^1507^AEWFLAYIL^1515^—derived from a transmembrane segment of the nsp3 protein of SARS-CoV-2—are fully buried within the antigen-binding cleft of HLA-B*40:01 (PDB ID: 6IEX, chain A). Moreover, the location of the partially solvent-exposed residues at Pos4-Pos8 (L1511, A1512, Y1513 and I1514) of the docked epitope along with solvent-exposed residues R62, T69, T73, E76, Q155, Y159, E163 and W167 of the α1 and α2 helices suggest an interaction of the ^1507^AEWFLAYIL^1515^–HLA-B*40:01 complex with TCR (Figure 2F).

## 4. Discussion

In order to tackle the current COVID-19 pandemic, it is critically important to better understand the underlying mechanism that gives rise to the individual differences in disease severity as well as to aid the vaccine development against the causative virus, SARS-CoV-2. Effective vaccinations are needed to eradicate the virus from populations all over the world and knowledge regarding the immunological response should have a significant impact on understanding disease progression. However, due to the limited experimental and clinical data currently available on the specific immune responses against SARS-CoV-2, the development of an effective vaccine against COVID-19 will be a challenge. This study sought to better understand the individual differences in the viral antigen presentation pathway and to aid the development of vaccines against COVID-19 by predicting in silico SARS-CoV-2 immunogenic epitopes.

Based solely on in silico predictions, the most potent SARS-CoV-2-derived MHC class I binding epitopes are ^1507^AEWFLAYIL^1515^ and ^1505^LVAEWFLAY^1513^ in terms of binding affinity, hydrophobicity and stability. However, for these “*in silico* epitopes”, only limited experimental data are available to correlate with. Therefore, in this study we mainly focused on potential SARS-CoV-2 epitopes that were conserved with SARS-CoV epitopes experimentally known to activate cytotoxic T-cells, and hence could be used in vaccine development.

The S glycoprotein-derived epitope ^1220^FIAGLIAIV^1228^ binds to the HLA-A*02:01 and HLA-A*02:06 allotypes with experimental IC_50_ values lower than 50 nM (Table 3). Our docking analysis supports these predictions, i.e., that epitope ^1220^FIAGLIAIV^1228^ could bind tightly to these allotypes and that ternary complexes with TCRs could form. Moreover, recent data demonstrate that patients with a severe form of COVID-19 have a stronger T-cell response after stimulation with the SARS-CoV-2 S-protein peptide pool compared to those with a mild manifestation of the disease [22,52]. The disease progression of COVID-19 is also associated with a higher magnitude of inflammatory cytokine-producing CD8+ T cells [52]. Whether these immune responses are due to strong binding of SARS-CoV-2 epitopes, including ^1220^FIAGLIAIV^1228^, to certain HLA allotypes, such as HLA-A*02:01 and HLA-A*02:06, or whether tight virus epitope-HLA interaction in general can actually be harmful for COVID-19 patients by causing, e.g., an immunological over-reaction, is not yet fully understood [53,54]. Furthermore, both CD4+ and CD8+ T-cells have been shown to be stimulated by overlapping peptides (15-mers overlapping by 10 amino acids) of the entire S glycoprotein sequence [55]. Does this mean that the S protein might function as a double-edged sword—that is, being crucial for viral entry into the host cell, but also important for overstimulating the immune responses, causing severe inflammation that aids the spread of the virus to surrounding cells? This still needs to be answered. The latter “sword” is known to be avoided at least by HIV, which has a sophisticated mechanism to limit the infection rate in order to better avoid immune surveillance [56,57].

A recent in vivo study shows that epitopes derived from the C-terminus of the S protein had a significantly stronger CD4+ T helper cell response in healthy donors in comparison to those infected with SARS-CoV-2 [22]. The CD4+ T cells’ cross-reactivity to the S protein might represent the key for understanding the different disease manifestations of COVID-19, particularly in the asymptomatic infections in children and adolescents. Our predicted epitope ^1220^FIAGLIAIV^1228^ overlaps with the C-terminal sequence of the S protein containing the S2 subunit, which is internalized after TMPRSS2 cleavage. Therefore, this particular amino acid sequence may also be important for inducing a protective immunological response towards immunity in COVID-19. However, canonically T helper cells recognize MHC class II molecules, whereas our prediction is based on the MHC class I molecules’ ability to present viral antigens for a possible cytotoxic T-cell response [58]. Indeed, we found four 15-mer epitopes that include the intact 9-mer ^1220^FIAGLIAIV^1228^ epitope sequence and showed binding affinities < 500 nM with DRB1*01:01 allotype of MHC class II (http://tools.iedb.org/mhcii/) [25], an allotype that is common in Caucasoid and Oriental ethnic backgrounds (https://www.ebi.ac.uk/ipd/imgt/hla/ethnicity.html) [59]. Nevertheless, there are a few reports regarding MHC class I-reactive CD4+ T helper cells, including the study where co-cultures of highly purified CD4+ T cells, together with a stimulatory MHC class II-negative cell line transfected with MHC class I molecules, were used to show the direct interaction of T helper cells with MHC class I molecules [60]. However, whether or not the ^1220^FIAGLIAIV^1228^ epitope is presented to both cytotoxic and helper T cells needs to be experimentally verified; a recent study that appeared while the current work was under review suggested that cross-reactivity could affect disease progression in COVID-19 [54]. Furthermore, the latest experimental reports suggest that the S glycoprotein of SARS-CoV-2 is both O- and *N*-glycosylated, especially on the RDB domain, which could mask immunogenic epitopes and may play an important role in SARS-CoV-2 immune evasion [61,62,63]. Fortunately, the predicted epitope ^1220^FIAGLIAIV^1228^ is part of a transmembrane helix, and consequently is neither O- nor *N*-glycosylated; the closest glycosylation site is at Pos1194, rendering this particular amino acid sequence potentially suitable for vaccine development.

Intriguingly, the SARS-CoV-2–derived, membrane glycoprotein epitope ^148^HLRIAGHHL^156^, which has low binding affinity (IC_50_ = 1693.63 nM; Table S4) towards HLA-B*15:02, is 78% identical in sequence with the intravirion SARS-CoV epitope HLRMAGHSL also from a membrane glycoprotein and shown to elicit a strong T-cell response in patients with the HLA-B*15:02 allotype [19]. Furthermore, HLA-B*15:02 has been shown to have a protective role against the severe forms of SARS-CoV [64]. This inspired us to do a separate in silico binding affinity analysis of the SARS-CoV HLRMAGHSL epitope to the HLA-B*15:02 allotype: a high binding affinity (IC_50_ = 232.85 nM) of the HLRMAGHSL epitope with the HLA-B*15:02 was predicted and agrees with the reported [19] protective immune response against SARS-CoV. Thus, there seems to be a difference between the highly conserved SARS-CoV-derived HLRMAGHSL and SARS-CoV-2-derived ^148^HLRIAGHHL^156^ epitopes in their potency towards the HLA-B*15:02 allotype. Moreover, the predicted low binding affinity of the SARS-CoV-2 epitopes with the HLA-B*15:02 allotype (Table S4) might not be sufficient to induce immune responses, thus rendering the potential of this specific epitope unfavorable for vaccination.

Based on our predictions, HLA-B*46:01 is one of the worst allotypes for presenting SARS-CoV-2-derived epitopes with an average binding score (IC_50_) of 2264 nM for the four epitopes predicted to bind (Table S4). This is in line with similar results from the predictions for SARS-CoV-2 and previous clinical data from SARS-CoV patients, demonstrating that this particular HLA allotype gives susceptibility to a more severe form of the viral disease [65]. Furthermore, our prediction shows that HLA-B*46:01 is not binding to either the S or M protein-derived peptides, strengthening the reported general view that HLA-B*46:01 is not optimal for eliciting an immune response in COVID-19 patients [58].

Taken together, identification of the predicted most immunogenic epitopes of SARS-CoV-2 could aid vaccine development. Since the sequences of the “top” epitopes of SARS-CoV-2 and SARS-CoV are highly conserved and the SARS epitopes are known to elicit an immunological response based on a previous study [66], a common vaccine protecting against both viruses and potential future strains is possible. Moreover, the selected experimentally known epitopes have been shown *not* to evoke an unwanted T cell cross-reactivity in vitro, further validating the potential use of the conserved SARS-CoV/CoV-2 peptides in vaccine development without disrupting self-tolerance [67]. However, there are many hurdles that need to be addressed; for example, due to the hydrophobic nature of these epitopes, they probably would need to be loaded in a liposome or nanocarrier for efficient vaccine delivery [68]. Moreover, as these conserved epitopes are presented by only a few HLA allotypes, i.e., A*02:01, A*02:06, A*68:02, which are common in American Indian, Caucasoid, Hispanic and Oriental ethnic backgrounds [59], the estimated world population coverage using these epitopes would only be around 42.1% (http://tools.iedb.org/population/) [25]. Therefore, developing a globally effective SARS-CoV-2 vaccine will probably require a pool of both novel and conserved epitopes, making a globally effective SARS-CoV-2 vaccine development a challenging task.

## 5. Conclusions

In the present study, we identified SARS-CoV-2 epitopes that were predicted to be presented by the MHC class I antigen processing pathway to the cytotoxic T cells. We report five purely in-silico-predicted most potent epitopes unique to SARS-CoV-2 and five potent SARS-CoV-2 epitope peptides identical to and experimentally determined for SARS-CoV. The novel SARS-CoV-2 epitopes were analyzed for their interaction with HLA allotypes using the IEDB and NetCTL1.2 web servers and three-dimensional structural models of selected, molecular dynamics simulation proven ternary complexes of SARS-CoV-2 pHLA–TCRs were created to assess interactions at structural level. HLA-A*02:01 and HLA-A*02:06 were found to have the greatest potential to present the selected epitopes, which are hydrophobic in nature and originated mainly from the transmembrane region of SARS-CoV-2 proteins. Our results could assist in the understanding of the individual and varying disease progression of COVID-19, as well as paving the way towards vaccine development against SARS-CoV-2.

## Figures and Tables

**Table 1:** SARS-CoV-2 proteins analyzed to predict major histocompatibility complex (MHC) class I binding epitopes.

Protein Name	Length (aa)	NCBI RefSeq Accession ID
nsp1	180	YP_009725297.1
nsp2	638	YP_009725298.1
nsp3	1945	YP_009725299.1
nsp4	500	YP_009725300.1
3C-like proteinase (3CLpro)	306	YP_009725301.1
nsp6	290	YP_009725302.1
nsp7	83	YP_009725303.1
nsp8	198	YP_009725304.1
nsp9	113	YP_009725305.1
nsp10	139	YP_009725306.1
nsp11	13	YP_009725312.1
RNA-dependent RNA polymerase (RdRp)	932	YP_009725307.1
Helicase	601	YP_009725308.1
3′-to-5′ exonuclease (35EXO)	527	YP_009725309.1
Endo RNAse (EndoR)	346	YP_009725310.1
2′-O-ribose methyltransferase	298	YP_009725311.1
Surface glycoprotein (S)	1273	YP_009724390.1
ORF3a	275	YP_009724391.1
Envelope protein (E)	75	YP_009724392.1
Membrane glycoprotein (M)	222	YP_009724393.1
ORF6	61	YP_009724394.1
ORF7a	121	YP_009724395.1
ORF7b	43	YP_009725318.1
ORF8	121	YP_009724396.1
Nucleocapsid phosphoprotein (N)	419	YP_009724397.2
ORF10	38	YP_009725255.1

**Table 2:** Most potent SARS-CoV-2–derived MHC class I binding epitopes identified with both the IEDB (lowest IC_50_) and NetCTL1.2 (highest combined score) in silico prediction methods.

Epitopes	Protein	Allotype	Supertype	Combined Score	Predicted IC_50_ (nM)
^738^DTDFVNEFY^746^	RdRp	A*01:01	A01	3.619	2.83
^1505^LVAEWFLAY^1513^	nsp3	A*29:02	A01	2.748	3.02
^289^SHFAIGLAL^297^	Helicase	B*39:01	B39	2.168	4.55
^1507^AEWFLAYIL^1515^	nsp3	B*40:01	B44	2.036	4.88
^1505^LVAEWFLAY^1513^	nsp3	B*35:01	A01	2.748	5.66
^1507^AEWFLAYIL^1515^	nsp3	B*40:02	B44	2.036	7.64
^217^AMDEFIERY^225^	EndoR	A*01:01	A01	3.138	10.47
^1505^LVAEWFLAY^1513^	nsp3	B*15:01	A01	2.748	11.16
^1505^LVAEWFLAY^1513^	nsp3	A*26:01	A01	2.748	18.88

**Table 3:** SARS-CoV-2–derived HLA-A*02 supertype-binding epitopes that are identical to the epitopes of SARS-CoV strains experimentally known to activate cytotoxic T-cells.

Epitopes	Protein	Epitope Mutation	Combined Score	Allotypes	Predicted IC_50_ (nM)	Experimental IC_50_ (nM)
^1220^FIAGLIAIV^1228^	S	No	1.212	A*02:01 A*02:06 A*68:02	10.29 11.13 8.32	1.48 2.8 0.54
^17^VLLFLAFVV^25^	E	No	1.213	A*02:01 A*02:06	21.72 107.83	5.62 12.6
^20^FLAFVVFLL^28^	E	No	1.440	A*02:01 A*02:06	5.26 51.99	0.23 2.57
^204^VLAWLYAAV^212^	3CLpro	No	1.173	A*02:01 A*02:06	13.40 29.50	0.435 8.79
^184^VLWAHGFEL^192^	35EXO	No	1.360	A*02:01 A*02:06	5.78 34.55	0.40 20.3
^330^LLSAGIFGA^338^	nsp3	I335V	1.217	A*02:01 A*02:06	10.09 14.54	8.1 24.6

**Table 4:** Predicted half-lives of complexes of the conserved SARS-CoV-2-derived most immunogenic experimentally identified epitopes and HLA-A*02 allotypes shown in Table 3. Secondary structures, localization within SARS-CoV-2 and GRAVY (grand average of hydropathicity index) scores of the epitopes.

Epitopes	Allotypes	Half-Life (in Hours)	Secondary Structure	Localization	GRAVY Score
^1220^FIAGLIAIV^1228^	A*02:01 A*02:06 A*68:02	5.11 5.42 0.63	helix	transmembrane	3.056
^17^VLLFLAFVV^25^	A*02:01 A*02:06	4.13 1.90	helix	transmembrane	3.489
^20^FLAFVVFLL^28^	A*02:01 A*02:06	11 5.48	helix	transmembrane	3.333
^204^VLAWLYAAV^212^	A*02:01 A*02:06	8.13 4.42	helix	intravirion	2.133
^184^VLWAHGFEL^192^	A*02:01 A*02:06	6.51 2.26	strand-coil-helix	intravirion	0.933

**Table 5:** Predicted half-lives of the novel SARS-CoV-2-derived, most immunogenic in-silico-identified epitopes in complex with the allotypes shown in Table 2. Secondary structures, localization within SARS-CoV-2, GRAVY scores and known mutations in the epitopes.

Epitopes	Allotype	Epitope Mutation	Half-Life (in Hours)	Localization	GRAVY Score
^738^DTDFVNEFY^746^	A*01:01	E744D T739I	2.84	intravirion	−0.689
^1505^LVAEWFLAY^1513^	A*29:02	No	3.64	transmembrane	1.389
^289^SHFAIGLAL^297^	B*39:01	H290Y A296S L297F	2.02	intravirion	1.567
^1507^AEWFLAYIL^1515^	B*40:01	No	2.04	transmembrane	1.422
^1505^LVAEWFLAY^1513^	B*35:01	No	1.69	transmembrane	1.389
^1507^AEWFLAYIL^1515^	B*40:02	No	3.81	transmembrane	1.422
^217^AMDEFIERY^225^	A*01:01	A217V F221L R224Q	1.26	intravirion	−0.589
^1505^LVAEWFLAY^1513^	B*15:01	No	7.51	transmembrane	1.389
^1505^LVAEWFLAY^1513^	A*26:01	No	1.33	transmembrane	1.389

**Table 6:** Comparison of the Cα atom positions of the five most immunogenic SARS-CoV-2 epitopes. Upper and lower triangular data show root–mean–square deviations in Ångströms for the epitopes in complex with HLA-A*02:01 and HLA-A*02:06, respectively.

	A*02:01	Fiagliaiv	Vllflafvv	Flafvvfll	Vlawlyaav	Vlwahgfel
A*02:06						
Fiagliaiv		0	0.685	0.566	0.554	0.852
Vllflafvv		0.940	0	0.65	0.881	0.699
Flafvvfll		1.002	0.636	0	0.518	0.888
Vlawlyaav		0.599	0.669	0.501	0	1.039
Vlwahgfel		1.296	1.250	1.470	1.432	0

## Supplementary Materials

The following material are available online at https://www.mdpi.com/2076-393X/8/3/408/s1, Figure S1: (A): Distribution of the predicted half-lives (log scale) of predicted (IEDB) 9-mer epitope-HLA-A*02 supertype complexes. The complexes are classified as immunogenic (IC50 ≤ 50 nM) and non-immunogenic (IC50 > 500 nM) based on the epitope binding affinity with the MHC molecule, (B): Distribution of the GRAVY scores of immunogenic (IC50 ≤ 50 nM) and non-immunogenic (IC50 > 500 nM) epitopes, Figure S1C and S1D: Comparison of the backbone conformation of the five epitopes docked into the cleft of HLA-A*02:01 (C) and HLA-A*02:06 (D) molecules against the canonical epitope 1GILGFVFTL9 of the influenza A virus (PDB ID: 5TEZ, chain C), Figure S2: Structural dynamics of the HLA-A*02:01-1220FIAGLIAIV1228 S protein epitope—TCR complex during three replicate 100 ns simulations. (A) Cα atom RMSF of the ternary complex. (B) Average Cα atom RMSF. Table S1A: MHC Class I allotypes association with supertypes described in published scientific literature [28,29]. Superscript “X” indicates availability of X-ray crystal structure in Protein Data Bank (PDB) [36], Table S1B: PDB codes for the X-ray crystal structures of MHC class I allotypes, Table S2: SARS-CoV-2–derived MHC class I binding epitopes identified with IEDB and NetCTL1.2 prediction methods as having strong binding affinity (IC50 ≤ 50 nM) with MHC molecules, Table S3: SARS-CoV-2-derived MHC class I binding epitopes identified with IEDB and NetCTL1.2 prediction methods as having weak binding affinity (50 nM < IC50 ≤ 500 nM) with MHC molecules, Table S4: SARS-CoV-2-derived MHC class I binding epitopes identified with IEDB and NetCTL1.2 prediction methods as having non-binding affinity (IC50 > 500 nM) with MHC molecules, Table S5: SARS-CoV-2-derived epitopes (immunogenicity score ≥ 0.25) and their prominent interacting HLA allotypes identified with IEDB and NetCTL1.2 prediction methods, Table S6: Most potent SARS-CoV-2-derived epitopes having immunogenicity score ≥ 0.25 and predicted binding affinity (IC50) ≤ 500 nM with their prominent interacting HLA allotypes identified with IEDB prediction method, Table S7: Most potent SARS-CoV-2-derived MHC class I binding epitopes identified with NetCTL1.2 (combined score ≥ 2) prediction method, Table S8: Most potent SARS-CoV-2-derived MHC class I binding epitopes that are identical to the experimentally known epitopes (MHC ligand assays data from the IEDB database) of SARS-CoV strains to activate cytotoxic T-cells. Predicted half-lives of the epitope—MHC-I complexes, GRAVY scores and mutations in the epitopes are shown. Table S9: Most frequently recorded intermolecular hydrogen bond interactions during the replicate 100 ns simulations.
