# Supplementary material for: Immunogenic SARS-CoV-2 Epitopes: In Silico Study Towards Better Understanding of COVID-19 Disease—Paving the Way for Vaccine Development
Source: Vaccines (Basel). 2020 Jul 23;8(3):408. doi: 10.3390/vaccines8030408 (PMC7564651; doi:10.3390/vaccines8030408)
Supplement: Supplementary file 1 [file vaccines-08-00408-s001.zip › Figure S1.pdf]

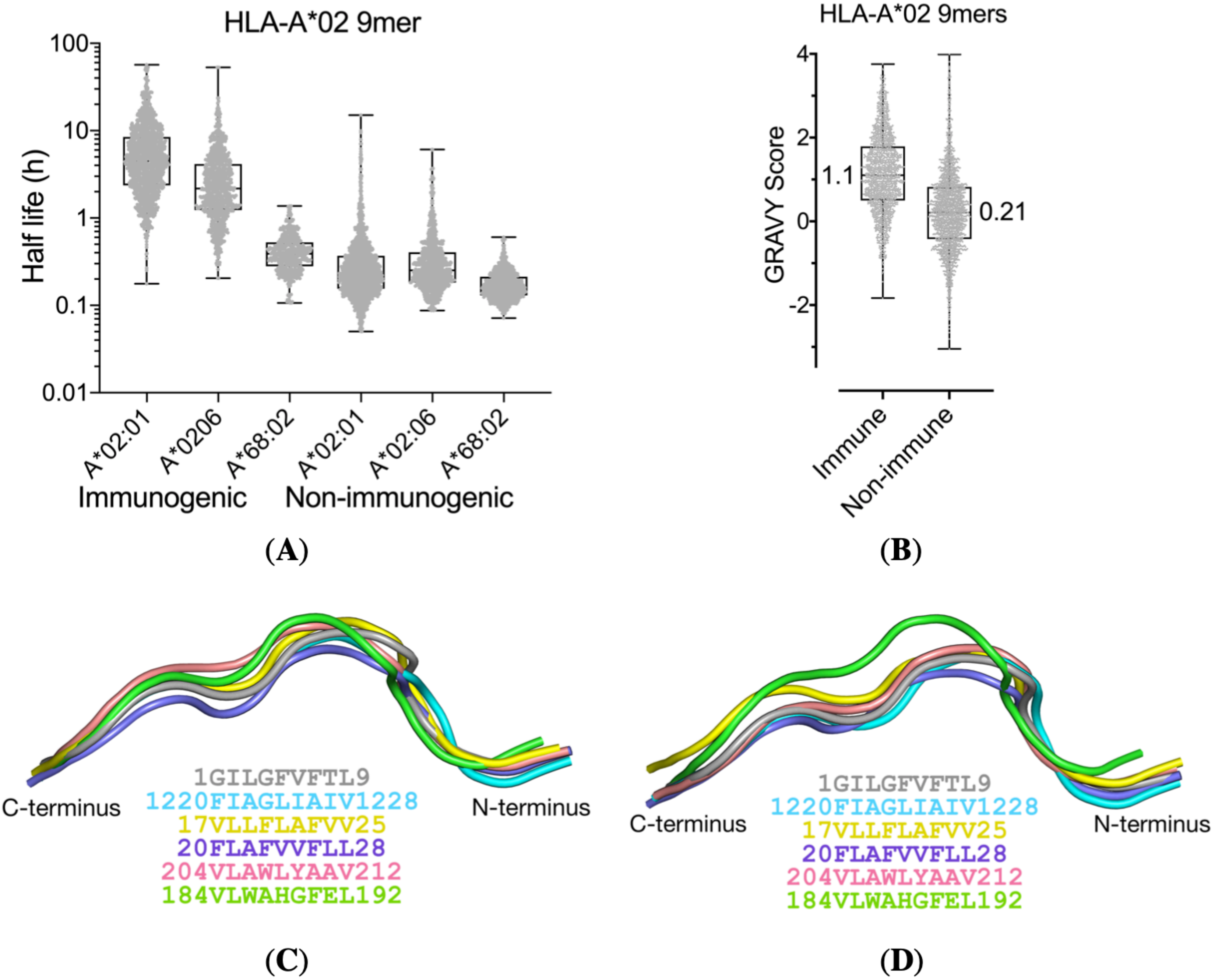

Supplementary Figure S1. (A) Distribution of the predicted half-lives (log scale) of predicted (IEDB) 9-mer epitope-HLA-A*02 supertype complexes. The complexes are classified as immunogenic ($IC_{50} \leq 50$ nM) and non- immunogenic ($IC_{50} > 500$ nM) based on the epitope binding affinity with the MHC molecule; (B) Distribution of the GRAVY scores of immunogenic ($IC_{50} \leq 50$ nM) and non-immunogenic ($IC_{50} > 500$ nM) epitopes; Comparison of backbone conformation of the five epitopes docked into the cleft of HLA-A*02:01 (C) and HLA-A*02:06 (D) molecules against the canonical epitope ^{1}GILGFVFTL^{9} of the influenza A virus (PDB ID: 5TEZ, chain C).
