# Supplementary material for: Immunogenic SARS-CoV-2 Epitopes: In Silico Study Towards Better Understanding of COVID-19 Disease—Paving the Way for Vaccine Development
Source: Vaccines (Basel). 2020 Jul 23;8(3):408. doi: 10.3390/vaccines8030408 (PMC7564651; doi:10.3390/vaccines8030408)
Supplement: Supplementary file 1 [file vaccines-08-00408-s001.zip › Figure S2.pdf]

(A)

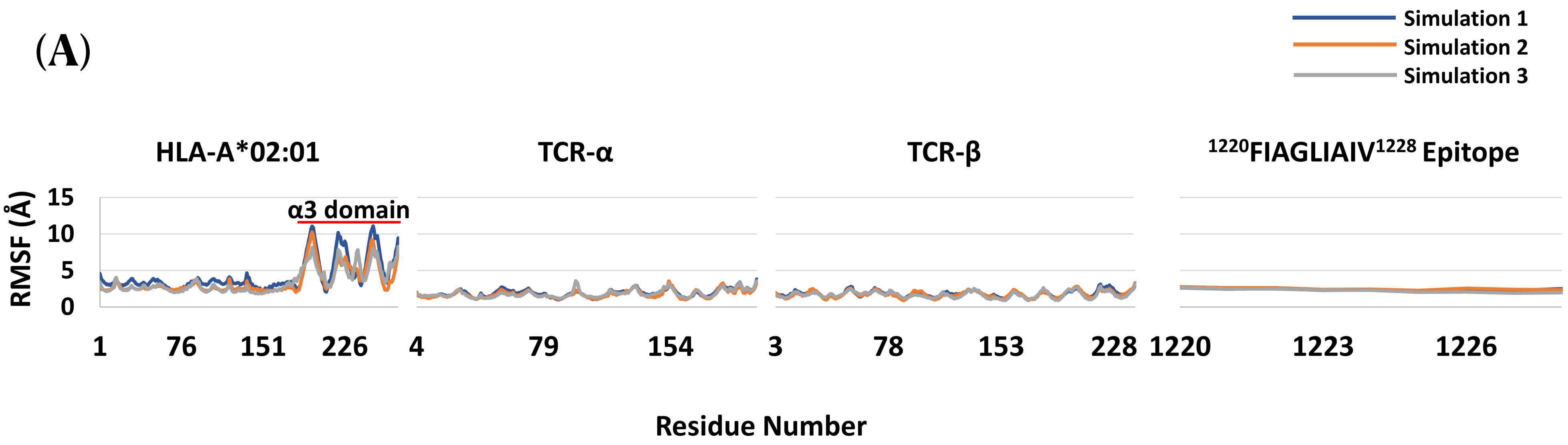

(B)

	Simulation 1 Average RMSF (Å)	Simulation 2 Average RMSF (Å)	Simulation 3 Average RMSF (Å)
HLA-A*02:01 – α1 domain	3.16 ± 0.48	2.64 ± 0.48	2.63 ± 0.44
HLA-A*02:01 – α2 domain	3.19 ± 0.46	2.49 ± 0.28	2.42 ± 0.47
HLA-A*02:01 – α3 domain	6.4 ± 2.57	5.32 ± 2.0	5.42 ± 1.45
TCR	1.86 ± 0.47	1.75 ± 0.47	1.75 ± 0.48
Epitope	2.36 ± 0.18	2.5 ± 0.16	2.25 ± 0.27

Supplementary Figure S2. Structural dynamics of the HLA-A*02:01–^{1220}FIAGLIAIV^{1228} S protein epitope–TCR complex during three replicate 100 ns simulations. (A) Cα atom RMSF of the ternary complex. (B) Average Cα atom RMSF.
