# Supplementary material for: Immunogenic SARS-CoV-2 Epitopes: In Silico Study Towards Better Understanding of COVID-19 Disease—Paving the Way for Vaccine Development
Source: Vaccines (Basel). 2020 Jul 23;8(3):408. doi: 10.3390/vaccines8030408 (PMC7564651; doi:10.3390/vaccines8030408)
Supplement: Supplementary file 1 [file vaccines-08-00408-s001.zip › Table S1.pdf]

Table S1A: MHC Class I allotypes association with supertypes described in published scientific literature [28,29]. Superscript "X" indicates availability of X-ray crystal structure in Protein Data Bank (PDB) [36].

Supertype	Allotypes
A01	A*01:01^{X}, A*25:01, A*29:02, A*30:01^{X}, A*30:02, A*32:01
A02	A*02:01^{X}, A*02:06^{X}, A*68:02^{X}
A03	A*03:01^{X}, A*11:01^{X}, A*30:01^{X} , A*31:01, A*33:03^{X}, A*68:01^{X}, A*74:01
A24	A*23:01, A*24:02^{X}, A*29:02
A26	A*26:01
B07	B*07:02^{X}, B*35:01^{X}, B*35:03, B*51:01^{X}, B*53:01^{X}, B*55:01, B*56:01
B08	B*08:01^{X}
B27	B*14:02^{X}, B*27:02, B*27:05^{X}, B*48:01
B39	B*38:01, B*39:01^{X}
B44	B*18:01^{X}, B*37:01, B*40:01^{X}, B*40:02^{X}, B*44:02^{X}, B*44:03^{X}, B*49:01, B*50:01
B58	B*57:01^{X}, B*58:01^{X}, B*58:02
B62	B*13:01, B*13:02, B*15:01^{X}, B*15:02, B*15:25, B*46:01^{X}, B*52:01^{X}

Table S1B: PDB codes for the X-ray crystal structures of MHC class I allotypes.

Allotype	PDB IDs	Allotype	PDB IDs
A*01:01	4NQX	B*18:01	4XXC
A*02:01	5TEZ, 5F9J, 6NCA, 6G3K, 6O4Y	B*27:05	5IB1, 2A83
A*02:06	3OXR	B*35:01	4LNR, 4PR5
A*03:01	3RL1, 6O9B, 6O9C	B*39:01	4O2E
A*08:01	6P23, 6P27, 6P2C, 6P2F, 6P2S	B*40:01	6IEX
A*11:01	6JP3, 6JOZ	B*40:02	5IEH
A*24:02	3WL9, 3WLB	B*44:02	1M6O
A*30:01	6J1W	B*44:03	1SYS
A*30:03	6J1V, 6J2A, 6J29	B*46:01	4LCY
A*68:01	6PBH	B*51:01	1E28, 1E27
B*07:02	6AT5	B*52:01	3W39
B*08:01	1M05	B*53:01	1A1M, 1A1O
B*14:02	3BVN	B*57:01	3X12, 2RFX, 2RFX, 5T6Y
B*15:01	1XR8	B*58:01	5VWH
