# Supplementary material for: Immunogenic SARS-CoV-2 Epitopes: In Silico Study Towards Better Understanding of COVID-19 Disease—Paving the Way for Vaccine Development
Source: Vaccines (Basel). 2020 Jul 23;8(3):408. doi: 10.3390/vaccines8030408 (PMC7564651; doi:10.3390/vaccines8030408)
Supplement: Supplementary file 1 [file vaccines-08-00408-s001.zip › Table S2.pdf]

Table S2: SARS-CoV-2–derived MHC class I binding epitopes identified with IEDB and NetCTL1.2 prediction methods as having strong binding affinity ($IC_{50} \leq 50$ nM) with MHC molecules.

Epitope	Allotype	IC_{50} (nM)
FVAAIFYLI	HLA-A*68:02	2.01
VPFWITIAY	HLA-B*35:01	2.43
SFYEDFLEY	HLA-A*29:02	2.73
DTDFVNEFY	HLA-A*01:01	2.83
YILFTRFFY	HLA-A*29:02	2.86
LVAEWFLAY	HLA-A*29:02	3.02
ILFTRFFYV	HLA-A*02:01	3.15
RMYIFFASF	HLA-A*32:01	4
ILFTRFFYV	HLA-A*02:06	4.27
GEVITFDNL	HLA-B*40:01	4.38
FLFVAAIFY	HLA-A*29:02	4.52
SHFAIGLAL	HLA-B*39:01	4.55
AEWFLAYIL	HLA-B*40:01	4.88
HFYWFFSNY	HLA-A*29:02	4.90
QWSLFFFLY	HLA-A*29:02	4.99
FLAFVVFLL	HLA-A*02:01	5.26
FVAAIFYLI	HLA-A*02:06	5.29
KLIEYTDFA	HLA-A*02:01	5.37
HVTFFIYNK	HLA-A*68:01	5.42
FELEDFIPM	HLA-B*18:01	5.48
RMYIFFASF	HLA-B*15:01	5.53
LVAEWFLAY	HLA-B*35:01	5.66
NVFAFPFTI	HLA-A*68:02	5.71
TEVVGDIIL	HLA-B*40:01	5.78
VLWAHGFEL	HLA-A*02:01	5.78
SPRWYFYYL	HLA-B*07:02	6.32
WLMWLIINL	HLA-A*02:01	6.60
KLIEYTDFA	HLA-A*02:06	7.14
AEWFLAYIL	HLA-B*40:02	7.64

Epitope	Allotype	IC_{50} (nM)
FLARGIVFM	HLA-A*02:01	7.71
NVLAWLYAA	HLA-A*02:06	8.31
FIAGLIAIV	HLA-A*68:02	8.32
FSYFAVHFI	HLA-A*68:02	8.38
FELEDFIPM	HLA-B*40:01	8.64
GTHWFVTQR	HLA-A*31:01	8.87
WPWYIWLGF	HLA-B*35:01	8.91
FLNRFTTTL	HLA-A*02:01	9.14
HVGEIPVAY	HLA-B*35:01	9.36
FELEDFIPM	HLA-A*02:06	9.78
LLSAGIFGA	HLA-A*02:01	10.09
MPYFFTLLL	HLA-B*53:01	10.13
TSAFVETVK	HLA-A*68:01	10.14
FIAGLIAIV	HLA-A*02:01	10.29
AMDEFIERY	HLA-A*01:01	10.47
FSSEIIGYK	HLA-A*68:01	10.85
LVSDIDITF	HLA-B*35:01	10.85
FIAGLIAIV	HLA-A*02:06	11.13
LVAEWFLAY	HLA-B*15:01	11.16
LVIGAVILR	HLA-A*68:01	11.41
RFRRAFGEY	HLA-A*30:01	11.41
MPYFFTLLL	HLA-B*35:01	11.75
RSFIEDLLF	HLA-B*58:01	11.93
FVAAIFYLI	HLA-A*02:01	11.96
VEHVTFFIY	HLA-B*18:01	11.99
MKIILFLAL	HLA-B*39:01	12.32
LTRNPAWRK	HLA-A*30:01	12.41
FLRDGWEIV	HLA-A*02:06	12.66
MLIIFWFSL	HLA-A*02:01	13.26
LMWLIINLV	HLA-A*02:01	13.29

Epitope	Allotype	IC_{50} (nM)
VPWDTIANY	HLA-B*35:01	13.33
VLAWLYAAV	HLA-A*02:01	13.40
SPRWYFYYL	HLA-B*08:01	13.77
HFAWWTAFV	HLA-A*68:02	13.91
EHFIETISL	HLA-B*39:01	13.95
LSYGIATVR	HLA-A*68:01	14.11
LLSAGIFGA	HLA-A*02:06	14.54
KSVNITFEL	HLA-B*58:01	15.38
MLIIFWFSL	HLA-A*32:01	15.86
IQYIDIGNY	HLA-A*30:02	15.96
WLTNIFGTV	HLA-A*02:06	16.12
EVVGDIILK	HLA-A*68:01	16.48
FQVTIAEIL	HLA-B*39:01	16.49
VFAFPFTIY	HLA-A*29:02	16.71
HVTFFIYNK	HLA-A*11:01	17.32
DLSPRWYFY	HLA-A*29:02	17.50
RSFIEDLLF	HLA-B*57:01	17.84
FQVTIAEIL	HLA-A*02:06	18.44
LVAEWFLAY	HLA-A*26:01	18.88
KVSIWNLDY	HLA-A*29:02	20.21
KLIFLWLLW	HLA-A*32:01	20.27
IFLWLLWPV	HLA-A*02:06	20.30
VFLFVAAIF	HLA-A*23:01	20.39
QHEETIYNL	HLA-B*39:01	20.70
SLREVRTIK	HLA-A*30:01	21.05
VLLFLAFVV	HLA-A*02:01	21.72
LAAECTIFK	HLA-A*68:01	22.02
YINVFAFPF	HLA-A*32:01	22.11
GTHWFVTQR	HLA-A*68:01	22.31
LLDDFVEII	HLA-A*02:01	23.67

Epitope	Allotype	IC_{50} (nM)
FLIVAAIVF	HLA-B*15:01	24
MGYINVFAF	HLA-B*35:01	24.08
TQLGIEFLK	HLA-A*11:01	24.55
YILFTRFFY	HLA-A*30:02	24.64
KLIFLWLLW	HLA-B*58:01	24.75
LSYGIATVR	HLA-A*31:01	24.87
LAAECTIFK	HLA-A*11:01	25.46
MLIIFWFSL	HLA-A*02:06	25.51
LVAEWFLAY	HLA-A*01:01	26.28
NVFAFPFTI	HLA-A*32:01	26.38
YINVFAFPF	HLA-B*35:01	26.67
LIVAAIVFI	HLA-A*02:06	26.90
ITSGWTFGA	HLA-A*68:02	27.59
FLARGIVFM	HLA-A*02:06	28.07
SELVIGAVI	HLA-B*40:01	28.08
KLMGHFAWW	HLA-A*32:01	29.42
VLAWLYAAV	HLA-A*02:06	29.50
AEWFLAYIL	HLA-B*44:03	29.53
LSPRWYFYY	HLA-A*29:02	29.94
STNVTIATY	HLA-A*30:02	30.16
WLMWLIINL	HLA-A*02:06	31.04
KLFIRQEEV	HLA-A*02:01	31.81
LTNIFGTVY	HLA-A*01:01	31.99
FLFLTWICL	HLA-A*02:01	32.26
WEPEFYEAM	HLA-B*18:01	32.40
VPWDTIANY	HLA-B*53:01	33.04
KLINIIIWF	HLA-A*32:01	33.93
VLWAHGFEL	HLA-A*02:06	34.55
HYVRITGLY	HLA-A*29:02	35.21
VTWFHAIHV	HLA-A*68:02	35.91

Epitope	Allotype	IC_{50} (nM)
LTNIFGTVY	HLA-B*15:01	36.49
DYGARFYFY	HLA-A*29:02	38.02
AANTVIWDY	HLA-A*30:02	39
HFYWFFSNY	HLA-A*30:02	39.02
FSSEIIGYK	HLA-A*11:01	39.17
VPFWITIAY	HLA-B*18:01	39.28
WEPEFYEAM	HLA-B*40:01	40.36
FMRFRRAFG	HLA-B*08:01	41.75
FLRDGWEIV	HLA-A*02:01	42.13
WPWYIWLGF	HLA-B*53:01	42.30
FELEDFIPM	HLA-B*40:02	42.48
HYVRITGLY	HLA-A*30:02	43.01
SELVIGAVI	HLA-B*40:02	43.47
QIGEYTFEK	HLA-A*11:01	43.73
MPYFFTLLL	HLA-B*39:01	44.17
ATAEAELAK	HLA-A*11:01	46.33
TSAFVETVK	HLA-A*11:01	46.54
VEHVTFFIY	HLA-B*44:03	47.43
FQVTIAEIL	HLA-B*40:01	47.55
TLADAGFIK	HLA-A*11:01	48.34
LSPRWYFYY	HLA-A*01:01	48.64
NIALIWNVK	HLA-A*68:01	49.44
