# Supplementary material for: Immunogenic SARS-CoV-2 Epitopes: In Silico Study Towards Better Understanding of COVID-19 Disease—Paving the Way for Vaccine Development
Source: Vaccines (Basel). 2020 Jul 23;8(3):408. doi: 10.3390/vaccines8030408 (PMC7564651; doi:10.3390/vaccines8030408)
Supplement: Supplementary file 1 [file vaccines-08-00408-s001.zip › Table S3.pdf]

Table S3: SARS-CoV-2–derived MHC class I binding epitopes identified with IEDB and NetCTL1.2 prediction methods as having weak binding affinity (50 nM < IC_{50} ≤ 500 nM) with MHC molecules.

Epitope	Allotype	IC_{50} (nM)
MFLARGIVF	HLA-A*23:01	50.53
FGDDTVIEV	HLA-A*02:06	51.78
FLAFVVFLL	HLA-A*02:06	51.99
MPYFFTLLL	HLA-B*07:02	52.87
VFVLWAHGF	HLA-A*23:01	53.56
TLRVEAFEY	HLA-A*29:02	55.46
CVDIPGIPK	HLA-A*11:01	55.47
AEWFLAYIL	HLA-B*18:01	56.75
LFVAAIFYL	HLA-A*23:01	57.15
KLMGHFAWW	HLA-B*58:01	57.81
IQYIDIGNY	HLA-B*15:01	59.22
SHFAIGLAL	HLA-B*38:01	60.43
FVAAIFYLI	HLA-A*29:02	60.61
AYILFTRFF	HLA-A*24:02	60.62
FELEDFIPM	HLA-B*35:01	60.81
TLADAGFIK	HLA-A*68:01	60.95
IMRLWLCWK	HLA-A*03:01	61.10
RMYIFFASF	HLA-A*23:01	62.24
RMYIFFASF	HLA-A*24:02	62.24
STNVTIATY	HLA-B*15:01	62.85
IVAGGIVAI	HLA-A*68:02	63.63
AYILFTRFF	HLA-A*23:01	64.51
LTRNPAWRK	HLA-A*03:01	67.10
SLREVRTIK	HLA-A*03:01	67.66
KVSIWNLDY	HLA-A*30:02	68.51
FWITIAYII	HLA-A*23:01	68.87
VVYRAFDIY	HLA-A*30:02	70.20
FLFVAAIFY	HLA-B*35:01	71.25
LSPRWYFYY	HLA-A*30:02	74.89

Epitope	Allotype	IC_{50} (nM)
REHEHEIAW	HLA-B*44:03	75.78
RFRRAFGEY	HLA-A*30:02	79.67
LMWLIINLV	HLA-A*02:06	80.33
VFLFVAAIF	HLA-A*24:02	80.73
IVAGGIVAI	HLA-A*32:01	80.74
LLEDEFTPF	HLA-B*15:01	81.14
SELVIGAVI	HLA-B*44:03	82.78
YINVFAFPF	HLA-B*15:01	83.83
IFLWLLWPV	HLA-A*02:01	87.56
HVGEIPVAY	HLA-A*29:02	89
KEGFFTYIC	HLA-B*40:02	95.50
FVAAIFYLI	HLA-B*53:01	96.72
YRINWITGG	HLA-B*27:05	98.22
FWITIAYII	HLA-A*24:02	98.54
GEVITFDNL	HLA-B*40:02	101.31
VFAFPFTIY	HLA-A*30:02	101.96
HVTFFIYNK	HLA-A*30:01	104.38
VLLFLAFVV	HLA-A*02:06	107.83
KRVDWTIEY	HLA-B*27:05	110.62
FLAFVVFLL	HLA-A*68:02	111.56
FLNRFTTTL	HLA-B*08:01	114.81
STKHFYWFF	HLA-A*26:01	117.86
MLIIFWFSL	HLA-B*08:01	118.70
HVTFFIYNK	HLA-A*03:01	119.02
VPHVGEIPV	HLA-B*07:02	119.11
GYAFEHIVY	HLA-A*29:02	120.67
VPFWITIAY	HLA-B*53:01	120.82
AEWFLAYIL	HLA-B*44:02	122.31
SQSIIAYTM	HLA-B*39:01	123.89
FELEDFIPM	HLA-B*39:01	125.67

Epitope	Allotype	IC_{50} (nM)
LAYILFTRF	HLA-B*35:01	132.38
FSYFAVHFI	HLA-B*58:01	133.13
STKHFYWFF	HLA-A*24:02	136.03
LAAECTIFK	HLA-A*03:01	142.36
VFVLWAHGF	HLA-A*24:02	142.61
WEPEFYEAM	HLA-B*40:02	144.78
VPFWITIAY	HLA-A*29:02	148.55
MKIILFLAL	HLA-B*08:01	153.52
LTNIFGTVY	HLA-A*29:02	155.95
IIFWFSLEL	HLA-A*32:01	157.05
FLNRFTTTL	HLA-B*39:01	159
LAYILFTRF	HLA-B*53:01	159.25
FLFLTWICL	HLA-B*39:01	161.22
MPYFFTLLL	HLA-B*51:01	162.35
STNVTIATY	HLA-A*01:01	164.70
KLIFLWLLW	HLA-B*57:01	170.58
LFLTWICLL	HLA-A*23:01	171.35
RMYIFFASF	HLA-B*08:01	172.56
FLFVAAIFY	HLA-B*15:01	179.41
KLIFLWLLW	HLA-A*23:01	182.96
MGYINVFAF	HLA-A*23:01	183.80
ETTADIVVF	HLA-A*26:01	187.40
PWYIWLGFI	HLA-A*23:01	187.82
FRLFARTRS	HLA-B*27:05	188.55
LTNIFGTVY	HLA-A*30:02	188.59
QHEETIYNL	HLA-B*38:01	196.16
LAYILFTRF	HLA-B*58:01	197.11
TLADAGFIK	HLA-A*03:01	198.58
LAYILFTRF	HLA-B*15:01	198.91
QWSLFFFLY	HLA-A*30:02	201.44

Epitope	Allotype	IC_{50} (nM)
REHEHEIAW	HLA-B*44:02	218.73
MRNAGIVGV	HLA-B*39:01	219.69
AANTVIWDY	HLA-B*35:01	220.60
RFRRAFGEY	HLA-A*29:02	221.20
MFLARGIVF	HLA-A*24:02	223.67
HFYWFFSNY	HLA-B*35:01	235.65
IFLWLLWPV	HLA-A*23:01	236.36
STNVTIATY	HLA-A*26:01	242.09
MRIFTIGTV	HLA-B*39:01	252.02
FQVTIAEIL	HLA-B*48:01	258.85
KLMGHFAWW	HLA-B*57:01	268.05
ILFTRFFYV	HLA-B*08:01	271.11
DLSPRWYFY	HLA-A*30:02	275.95
WNLVIGFLF	HLA-A*23:01	277.26
FVAAIFYLI	HLA-A*23:01	285.02
DSKEGFFTY	HLA-B*18:01	294.47
GHFAWWTAF	HLA-B*38:01	300.95
RMYIFFASF	HLA-B*48:01	303.18
EHYVRITGL	HLA-B*39:01	325.84
DGARRVWTL	HLA-B*14:02	326.18
LIMLIIFWF	HLA-A*23:01	328.50
KVSIWNLDY	HLA-A*01:01	333.91
RHVRAWIGF	HLA-A*24:02	362.97
MFLARGIVF	HLA-B*35:01	365.15
LFTRFFYVL	HLA-A*23:01	382.15
QQWGFTGNL	HLA-B*48:01	384.53
EHFIETISL	HLA-B*38:01	397.15
AIVFITLCF	HLA-A*32:01	399.10
FVAAIFYLI	HLA-A*32:01	417.26
KLIFLWLLW	HLA-A*24:02	428.13

Epitope	Allotype	IC_{50} (nM)
YINVFAFPF	HLA-B*53:01	440.85
MPYFFTLLL	HLA-B*08:01	454.87
DSKEGFFTY	HLA-A*26:01	458.91
LEGYAFEHI	HLA-B*40:01	463.52
RSFIEDLLF	HLA-A*32:01	465.90
EEEQEEDWL	HLA-B*40:01	490.18
SELVIGAVI	HLA-B*44:02	496.94
