# Supplementary material for: Immunogenic SARS-CoV-2 Epitopes: In Silico Study Towards Better Understanding of COVID-19 Disease—Paving the Way for Vaccine Development
Source: Vaccines (Basel). 2020 Jul 23;8(3):408. doi: 10.3390/vaccines8030408 (PMC7564651; doi:10.3390/vaccines8030408)
Supplement: Supplementary file 1 [file vaccines-08-00408-s001.zip › Table S4.pdf]

Table S4: SARS-CoV-2–derived MHC class I binding epitopes identified with IEDB and NetCTL1.2 prediction methods as having non-binding affinity (IC_{50} > 500 nM) with MHC molecules.

Epitope	Allotype	IC_{50} (nM)
SHFAIGLAL	HLA-B*14:02	541.90
SHFAIGLAL	HLA-B*48:01	545.34
LIMLIIFWF	HLA-A*32:01	563.42
LIMLIIFWF	HLA-A*24:02	636.74
LAYILFTRF	HLA-B*51:01	720.85
NGDVVAIDY	HLA-A*01:01	731.96
IIKLIFLWL	HLA-B*08:01	751.39
GHFAWWTAF	HLA-B*14:02	778.42
FIAGLIAIV	HLA-A*26:01	802.95
FSYFAVHFI	HLA-B*51:01	805.33
FLFLTWICL	HLA-B*15:02	871.83
FSTFEEAAL	HLA-B*35:03	886.74
AEWFLAYIL	HLA-B*48:01	897.37
AEWFLAYIL	HLA-A*32:01	910.69
MKIILFLAL	HLA-B*48:01	928.43
GWLIVGVAL	HLA-B*48:01	933.14
DSKEGFFTY	HLA-A*25:01	935.74
MGYINVFAF	HLA-A*32:01	1058.26
HYVRITGLY	HLA-A*26:01	1091.74
FLARGIVFM	HLA-B*15:02	1332.02
FLIVAAIVF	HLA-B*46:01	1354.82
FLARGIVFM	HLA-A*26:01	1366.96
FLNRFTTTL	HLA-B*15:02	1407.69
ETTADIVVF	HLA-A*25:01	1450.38
FLFLTWICL	HLA-B*35:03	1502.71
STNVTIATY	HLA-A*25:01	1533.89
WLMWLIINL	HLA-B*38:01	1676.36
HLRIAGHHL	HLA-B*15:02	1693.63
FVAAIFYLI	HLA-A*26:01	1727.47

Epitope	Allotype	IC_{50} (nM)
LAYILFTRF	HLA-B*14:02	1846.78
MGYINVFAF	HLA-B*46:01	1869.59
HFYWFFSNY	HLA-A*26:01	2112.67
MPYFFTLLL	HLA-B*35:03	2254.42
FVAAIFYLI	HLA-B*51:01	2475.12
RNAGIVGVL	HLA-B*48:01	2528.11
YINVFAFPF	HLA-B*46:01	2607.09
FWITIAYII	HLA-B*51:01	2686.47
MPYFFTLLL	HLA-B*48:01	2777.68
FMRFRRAFG	HLA-B*14:02	2778.91
RMYIFFASF	HLA-B*46:01	3225.58
TVSWNLREM	HLA-A*25:01	3888.80
SQSIIAYTM	HLA-B*48:01	4697.12
SPRWYFYYL	HLA-B*35:03	4757.16
RRATRRIRG	HLA-B*14:02	5163.37
FIAGLIAIV	HLA-A*25:01	5479.67
FKNLREFVF	HLA-B*35:03	6932.52
TIAEILLII	HLA-A*25:01	7293.31
KLINIIIWF	HLA-B*58:02	7494.17
ETTADIVVF	HLA-B*58:02	8518.61
KSVNITFEL	HLA-B*58:02	11592.74
TSRYWEPEF	HLA-B*58:02	13562.21
