# Supplementary material for: Immunogenic SARS-CoV-2 Epitopes: In Silico Study Towards Better Understanding of COVID-19 Disease—Paving the Way for Vaccine Development
Source: Vaccines (Basel). 2020 Jul 23;8(3):408. doi: 10.3390/vaccines8030408 (PMC7564651; doi:10.3390/vaccines8030408)
Supplement: Supplementary file 1 [file vaccines-08-00408-s001.zip › Table S5.pdf]

Table S5: SARS-CoV-2–derived epitopes (immunogenicity score ≥ 0.25) and their prominent interacting HLA allotypes identified with IEDB and NetCTL1.2 prediction methods.

Epitopes	Protein	Allotypes
FIAGLIAIV	Surface glycoprotein	HLA-A*02:01; HLA-A*02:06; HLA-A*25:01; HLA-A*26:01; HLA-A*68:02
FKNLREFVF	Surface glycoprotein	HLA-B*35:03
GTHWFVTQR	Surface glycoprotein	HLA-A*31:01; HLA-A*33:03; HLA-A*68:01; HLA-A*74:01
ITSGWTFGA	Surface glycoprotein	HLA-A*68:02
KRSFIEDLL	Surface glycoprotein	HLA-B*27:02
PWYIWLGFI	Surface glycoprotein	HLA-A*23:01
QLTPTWRVY	Surface glycoprotein	HLA-B*15:25
QQLIRAAEI	Surface glycoprotein	HLA-B*13:01; HLA-B*13:02; HLA-B*37:01; HLA-B*52:01
RSFIEDLLF	Surface glycoprotein	HLA-A*32:01; HLA-B*13:01; HLA-B*13:02; HLA-B*15:25; HLA-B*52:01; HLA-B*57:01; HLA-B*58:01
SQSIIAYTM	Surface glycoprotein	HLA-B*13:01; HLA-B*13:02; HLA-B*15:25; HLA-B*37:01; HLA-B*39:01; HLA-B*48:01; HLA-B*52:01
TLADAGFIK	Surface glycoprotein	HLA-A*03:01; HLA-A*11:01; HLA-A*68:01; HLA-A*74:01
VTWFHAIHV	Surface glycoprotein	HLA-A*68:02; HLA-B*52:01
WPWYIWLGF	Surface glycoprotein	HLA-B*35:01; HLA-B*53:01
YECDIPIGA	Surface glycoprotein	HLA-B*13:02; HLA-B*37:01; HLA-B*49:01; HLA-B*50:01
DTDFVNEFY	RNA-dependent RNA polymerase	HLA-A*01:01
KFYGGWHNM	RNA-dependent RNA polymerase	HLA-B*13:01; HLA-B*15:25
MRNAGIVGV	RNA-dependent RNA polymerase	HLA-B*27:02; HLA-B*39:01
QHEETIYNL	RNA-dependent RNA polymerase	HLA-B*38:01; HLA-B*39:01
RNAGIVGVL	RNA-dependent RNA polymerase	HLA-B*13:01; HLA-B*48:01
SRYWEPEFY	RNA-dependent RNA polymerase	HLA-B*15:25; HLA-B*27:02
TSRYWEPEF	RNA-dependent RNA polymerase	HLA-B*58:02
VVYRAFDIY	RNA-dependent RNA polymerase	HLA-A*30:02; HLA-B*15:25
WEPEFYEAM	RNA-dependent RNA polymerase	HLA-B*13:01; HLA-B*18:01; HLA-B*37:01; HLA-B*40:01; HLA-B*40:02; HLA-B*49:01; HLA-B*50:01
IQYIDIGNY	ORF8	HLA-A*30:02; HLA-B*13:01; HLA-B*15:01; HLA-B*15:25; HLA-B*50:01; HLA-B*52:01
SFYEDFLEY	ORF8	HLA-A*29:02; HLA-B*15:25
VFLGIITTV	ORF8	HLA-B*52:01
IIFWFSLEL	ORF7b	HLA-A*32:01
LIMLIIFWF	ORF7b	HLA-A*23:01; HLA-A*24:02; HLA-A*32:01
MLIIFWFSL	ORF7b	HLA-A*02:01; HLA-A*02:06; HLA-A*32:01; HLA-B*08:01
AIVFITLCF	ORF7a	HLA-A*32:01
FLIVAAIVF	ORF7a	HLA-B*15:01; HLA-B*15:25; HLA-B*46:01
KLFIRQEEV	ORF7a	HLA-A*02:01
LIVAAIVFI	ORF7a	HLA-A*02:06
MKIILFLAL	ORF7a	HLA-B*08:01; HLA-B*39:01; HLA-B*48:01
SPIFLIVAA	ORF7a	HLA-B*55:01; HLA-B*56:01
VAAIVFITL	ORF7a	HLA-B*13:02; HLA-B*52:01; HLA-B*55:01; HLA-B*56:01
FQVTIAEIL	ORF6	HLA-A*02:06; HLA-B*13:01; HLA-B*13:02; HLA-B*37:01; HLA-B*39:01; HLA-B*40:01; HLA-B*48:01; HLA-B*52:01
KVSIWNLDY	ORF6	HLA-A*01:01; HLA-A*29:02; HLA-A*30:02; HLA-B*15:25
TIAEILLII	ORF6	HLA-A*25:01; HLA-B*52:01
GWLIVGVAL	ORF3a	HLA-B*48:01

Epitopes	Protein	Allotypes
HVTFFIYNK	ORF3a	HLA-A*03:01; HLA-A*11:01; HLA-A*30:01; HLA-A*33:03; HLA-A*68:01; HLA-A*74:01
IMRLWLCWK	ORF3a	HLA-A*03:01; HLA-A*74:01
MRIFTIGTV	ORF3a	HLA-B*27:02; HLA-B*39:01
TGVEHVTFF	ORF3a	HLA-B*52:01
VEHVTFFIY	ORF3a	HLA-B*18:01; HLA-B*44:03; HLA-B*49:01; HLA-B*50:01
MGYINVFAF	ORF10	HLA-A*23:01; HLA-A*32:01; HLA-B*15:25; HLA-B*35:01; HLA-B*46:01; HLA-B*52:01
NVFAFPFTI	ORF10	HLA-A*32:01; HLA-A*68:02; HLA-B*13:01; HLA-B*13:02; HLA-B*52:01; HLA-B*55:01; HLA-B*56:01
VFAFPFTIY	ORF10	HLA-A*29:02; HLA-A*30:02; HLA-B*15:25
YINVFAFPF	ORF10	HLA-A*32:01; HLA-B*15:01; HLA-B*35:01; HLA-B*46:01; HLA-B*53:01
DLSPRWYFY	Nucleocapsid phosphoprotein	HLA-A*29:02; HLA-A*30:02
LSPRWYFYY	Nucleocapsid phosphoprotein	HLA-A*01:01; HLA-A*29:02; HLA-A*30:02
RRATRRIRG	Nucleocapsid phosphoprotein	HLA-B*14:02
SPRWYFYYL	Nucleocapsid phosphoprotein	HLA-B*07:02; HLA-B*08:01; HLA-B*35:03; HLA-B*55:01; HLA-B*56:01
SALWEIQQV	nsp8	HLA-B*13:01; HLA-B*13:02; HLA-B*52:01
DGARRVWTL	nsp6	HLA-B*14:02
FLARGIVFM	nsp6	HLA-A*02:01; HLA-A*02:06; HLA-A*26:01; HLA-B*15:02; HLA-B*15:25
IFFITGNTL	nsp6	HLA-B*52:01
MFLARGIVF	nsp6	HLA-A*23:01; HLA-A*24:02; HLA-B*35:01
QWSLFFFLY	nsp6	HLA-A*29:02; HLA-A*30:02
AVITREVGF	nsp4	HLA-B*15:25
FLFVAAIFY	nsp4	HLA-A*29:02; HLA-B*15:01; HLA-B*15:25; HLA-B*35:01
FMRFRRAFG	nsp4	HLA-B*08:01; HLA-B*14:02
FSSEIIGYK	nsp4	HLA-A*11:01; HLA-A*33:03; HLA-A*68:01; HLA-A*74:01
FSTFEEAAL	nsp4	HLA-B*35:03
FVAAIFYLI	nsp4	HLA-A*02:01; HLA-A*02:06; HLA-A*23:01; HLA-A*26:01; HLA-A*29:02; HLA-A*32:01; HLA-A*68:02; HLA-B*51:01; HLA-B*53:01
FWITIAYII	nsp4	HLA-A*23:01; HLA-A*24:02; HLA-B*51:01
HFYWFFSNY	nsp4	HLA-A*26:01; HLA-A*29:02; HLA-A*30:02; HLA-B*35:01
IVAGGIVAI	nsp4	HLA-A*32:01; HLA-A*68:02; HLA-B*13:01; HLA-B*13:02; HLA-B*15:25; HLA-B*52:01; HLA-B*55:01; HLA-B*56:01
KLIEYTDFA	nsp4	HLA-A*02:01; HLA-A*02:06
LAAECTIFK	nsp4	HLA-A*03:01; HLA-A*11:01; HLA-A*68:01
LFVAAIFYL	nsp4	HLA-A*23:01
RFRRAFGEY	nsp4	HLA-A*29:02; HLA-A*30:01; HLA-A*30:02; HLA-B*15:25
STKHFYWFF	nsp4	HLA-A*24:02; HLA-A*26:01
VFLFVAAIF	nsp4	HLA-A*23:01; HLA-A*24:02
VPFWITIAY	nsp4	HLA-A*29:02; HLA-B*15:25; HLA-B*18:01; HLA-B*35:01; HLA-B*53:01; HLA-B*55:01; HLA-B*56:01
AEWFLAYIL	nsp3	HLA-A*32:01; HLA-B*13:01; HLA-B*13:02; HLA-B*18:01; HLA-B*37:01; HLA-B*40:01; HLA-B*40:02; HLA-B*44:02; HLA-B*44:03; HLA-B*48:01; HLA-B*49:01; HLA-B*50:01; HLA-B*52:01
ATAEAELAK	nsp3	HLA-A*11:01
AYILFTRFF	nsp3	HLA-A*23:01; HLA-A*24:02
DYGARFYFY	nsp3	HLA-A*29:02
EEEQEEDWL	nsp3	HLA-B*40:01

Epitopes	Protein	Allotypes
EHFIETISL	nsp3	HLA-B*38:01; HLA-B*39:01; HLA-B*52:01
EVVGDIILK	nsp3	HLA-A*68:01
FGDDTVIEV	nsp3	HLA-A*02:06; HLA-B*13:02; HLA-B*56:01
FSYFAVHFI	nsp3	HLA-A*68:02; HLA-B*13:02; HLA-B*51:01; HLA-B*52:01; HLA-B*58:01
GEVITFDNL	nsp3	HLA-B*13:01; HLA-B*13:02; HLA-B*37:01; HLA-B*40:01; HLA-B*40:02; HLA-B*49:01; HLA-B*50:01
HLDGEVITF	nsp3	HLA-B*13:01; HLA-B*13:02; HLA-B*15:25
ILFTRFFYV	nsp3	HLA-A*02:01; HLA-A*02:06; HLA-A*74:01; HLA-B*08:01; HLA-B*52:01
KLINIIIWF	nsp3	HLA-A*32:01; HLA-B*13:01; HLA-B*15:25; HLA-B*58:02
KSVNITFEL	nsp3	HLA-B*13:01; HLA-B*13:02; HLA-B*58:01; HLA-B*58:02
LAYILFTRF	nsp3	HLA-B*14:02; HLA-B*15:01; HLA-B*15:25; HLA-B*35:01; HLA-B*51:01; HLA-B*52:01; HLA-B*53:01; HLA-B*58:01
LFTRFFYVL	nsp3	HLA-A*23:01
LLSAGIFGA	nsp3	HLA-A*02:01; HLA-A*02:06
LMWLIINLV	nsp3	HLA-A*02:01; HLA-A*02:06
LRVEAFEYY	nsp3	HLA-B*27:02
LVAEWFLAY	nsp3	HLA-A*01:01; HLA-A*26:01; HLA-A*29:02; HLA-B*15:01; HLA-B*15:25; HLA-B*35:01
LVSDIDITF	nsp3	HLA-B*13:01; HLA-B*15:25; HLA-B*35:01; HLA-B*56:01
MPYFFTLLL	nsp3	HLA-B*07:02; HLA-B*08:01; HLA-B*35:01; HLA-B*35:03; HLA-B*39:01; HLA-B*48:01; HLA-B*51:01; HLA-B*52:01; HLA-B*53:01; HLA-B*55:01; HLA-B*56:01
NGDVVAIDY	nsp3	HLA-A*01:01
NIALIWNVK	nsp3	HLA-A*68:01
PEEHFIETI	nsp3	HLA-B*49:01
RMYIFFASF	nsp3	HLA-A*23:01; HLA-A*24:02; HLA-A*32:01; HLA-A*74:01; HLA-B*08:01; HLA-B*15:01; HLA-B*15:25; HLA-B*46:01; HLA-B*48:01; HLA-B*52:01
SLPINVIVF	nsp3	HLA-B*13:01; HLA-B*15:25
SLREVRTIK	nsp3	HLA-A*03:01; HLA-A*30:01; HLA-A*74:01
STNVTIATY	nsp3	HLA-A*01:01; HLA-A*25:01; HLA-A*26:01; HLA-A*30:02; HLA-B*15:01; HLA-B*15:25
TEVVGDIIL	nsp3	HLA-B*13:01; HLA-B*13:02; HLA-B*37:01; HLA-B*40:01; HLA-B*49:01; HLA-B*50:01
TLRVEAFEY	nsp3	HLA-A*29:02; HLA-B*15:25
TQLGIEFLK	nsp3	HLA-A*11:01; HLA-A*74:01
TVSWNLREM	nsp3	HLA-A*25:01
VPWDTIANY	nsp3	HLA-B*15:25; HLA-B*35:01; HLA-B*53:01; HLA-B*55:01; HLA-B*56:01
WLMWLIINL	nsp3	HLA-A*02:01; HLA-A*02:06; HLA-B*38:01
YILFTRFFY	nsp3	HLA-A*29:02; HLA-A*30:02; HLA-A*74:01
FLRDGWEIV	nsp2	HLA-A*02:01; HLA-A*02:06
KLNEEIAII	nsp2	HLA-B*13:01; HLA-B*13:02; HLA-B*52:01
LTNIFGTVY	nsp2	HLA-A*01:01; HLA-A*29:02; HLA-A*30:02; HLA-B*15:01; HLA-B*15:25
REHEHEIAW	nsp2	HLA-B*13:01; HLA-B*13:02; HLA-B*15:25; HLA-B*37:01; HLA-B*44:02; HLA-B*44:03; HLA-B*49:01; HLA-B*50:01; HLA-B*52:01
RKGGRTIAF	nsp2	HLA-B*15:25
TSAFVETVK	nsp2	HLA-A*11:01; HLA-A*68:01
WLTNIFGTV	nsp2	HLA-A*02:06
FLFLTWICL	Membrane glycoprotein	HLA-A*02:01; HLA-B*15:02; HLA-B*35:03; HLA-B*39:01

Epitopes	Protein	Allotypes
FRLFARTRS	Membrane glycoprotein	HLA-B*27:05
HLRIAGHHL	Membrane glycoprotein	HLA-B*15:02; HLA-B*15:25; HLA-B*55:01
IFLWLLWPV	Membrane glycoprotein	HLA-A*02:01; HLA-A*02:06; HLA-A*23:01
IIKLIFLWL	Membrane glycoprotein	HLA-B*08:01
KLIFLWLLW	Membrane glycoprotein	HLA-A*23:01; HLA-A*24:02; HLA-A*32:01; HLA-B*57:01; HLA-B*58:01
LFLTWICLL	Membrane glycoprotein	HLA-A*23:01
LPKEITVAT	Membrane glycoprotein	HLA-B*55:01; HLA-B*56:01
LVIGAVILR	Membrane glycoprotein	HLA-A*33:03; HLA-A*68:01; HLA-A*74:01
SELVIGAVI	Membrane glycoprotein	HLA-B*13:01; HLA-B*13:02; HLA-B*37:01; HLA-B*40:01; HLA-B*40:02; HLA-B*44:02; HLA-B*44:03; HLA-B*49:01; HLA-B*50:01; HLA-B*52:01
WNLVIGFLF	Membrane glycoprotein	HLA-A*23:01
YRINWITGG	Membrane glycoprotein	HLA-B*27:02; HLA-B*27:05
HVGEIPVAY	nsp1	HLA-A*29:02; HLA-B*15:25; HLA-B*35:01
VPHVGEIPV	nsp1	HLA-B*07:02; HLA-B*55:01; HLA-B*56:01
EHYVRITGL	Helicase	HLA-B*39:01; HLA-B*52:01
ETTADIVVF	Helicase	HLA-A*25:01; HLA-A*26:01; HLA-B*58:02
HYVRITGLY	Helicase	HLA-A*26:01; HLA-A*29:02; HLA-A*30:02
LSYGIATVR	Helicase	HLA-A*31:01; HLA-A*33:03; HLA-A*68:01; HLA-A*74:01
LTRNPAWRK	Helicase	HLA-A*03:01; HLA-A*30:01; HLA-A*74:01
PQIGVVREF	Helicase	HLA-B*13:01; HLA-B*15:25
QIGEYTFEK	Helicase	HLA-A*11:01; HLA-A*74:01
SHFAIGLAL	Helicase	HLA-B*13:01; HLA-B*13:02; HLA-B*14:02; HLA-B*27:02; HLA-B*37:01; HLA-B*38:01; HLA-B*39:01; HLA-B*48:01; HLA-B*49:01; HLA-B*50:01; HLA-B*52:01; HLA-B*55:01; HLA-B*56:01
FLAFVVFLL	Envelope protein	HLA-A*02:01; HLA-A*02:06; HLA-A*68:02
VLLFLAFVV	Envelope protein	HLA-A*02:01; HLA-A*02:06
AANTVIWDY	Endo RNAse	HLA-A*30:02; HLA-B*15:25; HLA-B*35:01
AMDEFIERY	Endo RNAse	HLA-A*01:01; HLA-B*13:01; HLA-B*15:25
FELEDFIPM	Endo RNAse	HLA-A*02:06; HLA-B*13:01; HLA-B*13:02; HLA-B*18:01; HLA-B*35:01; HLA-B*37:01; HLA-B*39:01; HLA-B*40:01; HLA-B*40:02; HLA-B*49:01; HLA-B*50:01; HLA-B*52:01
GYAFEHIVY	Endo RNAse	HLA-A*29:02
LEGYAFEHI	Endo RNAse	HLA-B*13:02; HLA-B*37:01; HLA-B*40:01; HLA-B*49:01; HLA-B*52:01
LLDDFVEII	Endo RNAse	HLA-A*02:01; HLA-B*13:02; HLA-B*52:01
AVINGDRWF	3C-like proteinase	HLA-B*13:01; HLA-B*15:25
FLNRFTTTL	3C-like proteinase	HLA-A*02:01; HLA-B*08:01; HLA-B*15:02; HLA-B*15:25; HLA-B*39:01
GSVGFNIDY	3C-like proteinase	HLA-B*15:25
LLEDEFTPF	3C-like proteinase	HLA-B*15:01; HLA-B*15:25
NVLAWLYAA	3C-like proteinase	HLA-A*02:06
VLAWLYAAV	3C-like proteinase	HLA-A*02:01; HLA-A*02:06
CVDIPGIPK	3′ to 5′ exonuclease	HLA-A*11:01
KRVDWTIEY	3′ to 5′ exonuclease	HLA-B*15:25; HLA-B*27:02; HLA-B*27:05
NLWNTFTRL	3′ to 5′ exonuclease	HLA-B*13:01
QQWGFTGNL	3′ to 5′ exonuclease	HLA-B*13:01; HLA-B*13:02; HLA-B*37:01; HLA-B*48:01; HLA-B*52:01
RHVRAWIGF	3′ to 5′ exonuclease	HLA-A*24:02; HLA-B*27:02
VFVLWAHGF	3′ to 5′ exonuclease	HLA-A*23:01; HLA-A*24:02

Epitopes	Protein	Allotypes
VLWAHGFEL	3′ to 5′ exonuclease	HLA-A*02:01; HLA-A*02:06; HLA-B*13:01; HLA-B*13:02; HLA-B*15:25; HLA-B*52:01
DSKEGFFTY	2′-O-ribose methyltransferase	HLA-A*25:01; HLA-A*26:01; HLA-B*15:25; HLA-B*18:01
GHFAWWTAF	2′-O-ribose methyltransferase	HLA-B*14:02; HLA-B*27:02; HLA-B*38:01; HLA-B*52:01
HFAWWTAFV	2′-O-ribose methyltransferase	HLA-A*33:03; HLA-A*68:02
KEGFFTYIC	2′-O-ribose methyltransferase	HLA-B*40:02
KLMGHFAWW	2′-O-ribose methyltransferase	HLA-A*32:01; HLA-B*13:01; HLA-B*57:01; HLA-B*58:01
