# Supplementary material for: Immunogenic SARS-CoV-2 Epitopes: In Silico Study Towards Better Understanding of COVID-19 Disease—Paving the Way for Vaccine Development
Source: Vaccines (Basel). 2020 Jul 23;8(3):408. doi: 10.3390/vaccines8030408 (PMC7564651; doi:10.3390/vaccines8030408)
Supplement: Supplementary file 1 [file vaccines-08-00408-s001.zip › Table S6.pdf]

Table S6: Most potent SARS-CoV-2–derived epitopes as having immunogenicity score ≥ 0.25 and binding affinity (IC50) ≤ 500 nM with their prominent interacting HLA allotypes identified with IEDB prediction method.

Epitopes	Start-End	Protein	Predicted allotypes	Binding affinity (IC_{50})
FVAAIFYLI	19-27	nsp4	HLA-A*68:02	2.01
VPFWITIAY	373-381	nsp4	HLA-B*35:01	2.43
SFYEDFLEY	103-111	ORF8	HLA-A*29:02	2.73
DTDFVNEFY	738-746	RNA-dependent RNA polymerase	HLA-A*01:01	2.83
YILFTRFFY	1513-1521	nsp3	HLA-A*29:02	2.86
LVAEWFLAY	1505-1513	nsp3	HLA-A*29:02	3.02
ILFTRFFYV	1514-1522	nsp3	HLA-A*02:01	3.15
RMYIFFASF	1564-1572	nsp3	HLA-A*32:01	4
ILFTRFFYV	1514-1522	nsp3	HLA-A*02:06	4.27
GEVITFDNL	730-738	nsp3	HLA-B*40:01	4.38
FLFVAAIFY	17-25	nsp4	HLA-A*29:02	4.52
SHFAIGLAL	289-297	Helicase	HLA-B*39:01	4.55
AEWFLAYIL	1507-1515	nsp3	HLA-B*40:01	4.88
HFYWFFSNY	389-397	nsp4	HLA-A*29:02	4.9
QWSLFFFLY	30-38	nsp6	HLA-A*29:02	4.99
FLAFVVFLL	20-28	Envelope protein	HLA-A*02:01	5.26
FVAAIFYLI	19-27	nsp4	HLA-A*02:06	5.29
KLIEYTDFA	138-146	nsp4	HLA-A*02:01	5.37
HVTFFIYNK	227-235	ORF3a	HLA-A*68:01	5.42
FELEDFIPM	263-271	Endo RNAse	HLA-B*18:01	5.48
RMYIFFASF	1564-1572	nsp3	HLA-B*15:01	5.53
LVAEWFLAY	1505-1513	nsp3	HLA-B*35:01	5.66
NVFAFPFTI	5-13	ORF10	HLA-A*68:02	5.71
TEVVGDIIL	1251-1259	nsp3	HLA-B*40:01	5.78
VLWAHGFEL	184-192	3′-to-5′ exonuclease	HLA-A*02:01	5.78
SPRWYFYYL	105-113	Nucleocapsid phosphoprotein	HLA-B*07:02	6.32
WLMWLIINL	1545-1553	nsp3	HLA-A*02:01	6.6
KLIEYTDFA	138-146	nsp4	HLA-A*02:06	7.14
AEWFLAYIL	1507-1515	nsp3	HLA-B*40:02	7.64
FLARGIVFM	184-192	nsp6	HLA-A*02:01	7.71
NVLAWLYAA	203-211	3C-like proteinase	HLA-A*02:06	8.31
FIAGLIAIV	1220-1228	Surface glycoprotein	HLA-A*68:02	8.32
FSYFAVHFI	1533-1541	nsp3	HLA-A*68:02	8.38
FELEDFIPM	263-271	Endo RNAse	HLA-B*40:01	8.64
GTHWFVTQR	1099-1107	Surface glycoprotein	HLA-A*31:01	8.87
WPWYIWLGF	1212-1220	Surface glycoprotein	HLA-B*35:01	8.91
FLNRFTTTL	219-227	3C-like proteinase	HLA-A*02:01	9.14
HVGEIPVAY	110-118	nsp1	HLA-B*35:01	9.36
FELEDFIPM	263-271	Endo RNAse	HLA-A*02:06	9.78
LLSAGIFGA	330-338	nsp3	HLA-A*02:01	10.09
MPYFFTLLL	1351-1359	nsp3	HLA-B*53:01	10.13
TSAFVETVK	304-312	nsp2	HLA-A*68:01	10.14
FIAGLIAIV	1220-1228	Surface glycoprotein	HLA-A*02:01	10.29
AMDEFIERY	217-225	Endo RNAse	HLA-A*01:01	10.47

Epitopes	Start-End	Protein	Predicted allotypes	Binding affinity (IC_{50})
FSSEIIGYK	39-47	nsp4	HLA-A*68:01	10.85
LVSDIDITF	452-460	nsp3	HLA-B*35:01	10.85
FIAGLIAIV	1220-1228	Surface glycoprotein	HLA-A*02:06	11.13
LVAEWFLAY	1505-1513	nsp3	HLA-B*15:01	11.16
LVIGAVILR	138-146	Membrane glycoprotein	HLA-A*68:01	11.41
RFRRAFGEY	303-311	nsp4	HLA-A*30:01	11.41
MPYFFTLLL	1351-1359	nsp3	HLA-B*35:01	11.75
RSFIEDLLF	815-823	Surface glycoprotein	HLA-B*58:01	11.93
FVAAIFYLI	19-27	nsp4	HLA-A*02:01	11.96
VEHVTFFIY	225-233	ORF3a	HLA-B*18:01	11.99
MKIILFLAL	1-9	ORF7a	HLA-B*39:01	12.32
LTRNPAWRK	500-508	Helicase	HLA-A*30:01	12.41
FLRDGWEIV	461-469	nsp2	HLA-A*02:06	12.66
MLIIFWFSL	24-32	ORF7b	HLA-A*02:01	13.26
LMWLIINLV	1546-1554	nsp3	HLA-A*02:01	13.29
VPWDTIANY	1315-1323	nsp3	HLA-B*35:01	13.33
VLAWLYAAV	204-212	3C-like proteinase	HLA-A*02:01	13.4
SPRWYFYYL	105-113	Nucleocapsid phosphoprotein	HLA-B*08:01	13.77
HFAWWTAFV	186-194	2′-O-ribosemethyl transferase	HLA-A*68:02	13.91
EHFIETISL	681-689	nsp3	HLA-B*39:01	13.95
LSYGIATVR	147-155	Helicase	HLA-A*68:01	14.11
LLSAGIFGA	330-338	nsp3	HLA-A*02:06	14.54
KSVNITFEL	19-27	nsp3	HLA-B*58:01	15.38
MLIIFWFSL	24-32	ORF7b	HLA-A*32:01	15.86
IQYIDIGNY	71-79	ORF8	HLA-A*30:02	15.96
WLTNIFGTV	432-440	nsp2	HLA-A*02:06	16.12
EVVGDIILK	1252-1260	nsp3	HLA-A*68:01	16.48
FQVTIAEIL	7-15	ORF6	HLA-B*39:01	16.49
VFAFPFTIY	6-14	ORF10	HLA-A*29:02	16.71
HVTFFIYNK	227-235	ORF3a	HLA-A*11:01	17.32
DLSPRWYFY	103-111	Nucleocapsid phosphoprotein	HLA-A*29:02	17.5
RSFIEDLLF	815-823	Surface glycoprotein	HLA-B*57:01	17.84
FQVTIAEIL	7-15	ORF6	HLA-A*02:06	18.44
LVAEWFLAY	1505-1513	nsp3	HLA-A*26:01	18.88
KVSIWNLDY	23-31	ORF6	HLA-A*29:02	20.21
KLIFLWLLW	50-58	Membrane glycoprotein	HLA-A*32:01	20.27
IFLWLLWPV	52-60	Membrane glycoprotein	HLA-A*02:06	20.3
VFLFVAAIF	16-24	nsp4	HLA-A*23:01	20.39
QHEETIYNL	81-89	RNA-dependent RNA polymerase	HLA-B*39:01	20.7
SLREVRTIK	743-751	nsp3	HLA-A*30:01	21.05
VLLFLAFVV	17-25	Envelope protein	HLA-A*02:01	21.72
LAAECTIFK	152-160	nsp4	HLA-A*68:01	22.02
YINVFAFPF	3-11	ORF10	HLA-A*32:01	22.11
GTHWFVTQR	1099-1107	Surface glycoprotein	HLA-A*68:01	22.31
LLDDFVEII	298-306	Endo RNAse	HLA-A*02:01	23.67

Epitopes	Start-End	Protein	Predicted allotypes	Binding affinity (IC_{50})
FLIVAAIVF	101-109	ORF7a	HLA-B*15:01	24
MGYINVFAF	1-9	ORF10	HLA-B*35:01	24.08
TQLGIEFLK	703-711	nsp3	HLA-A*11:01	24.55
YILFTRFFY	1513-1521	nsp3	HLA-A*30:02	24.64
KLIFLWLLW	50-58	Membrane glycoprotein	HLA-B*58:01	24.75
LSYGIATVR	147-155	Helicase	HLA-A*31:01	24.87
LAAECTIFK	152-160	nsp4	HLA-A*11:01	25.46
MLIIFWFSL	24-32	ORF7b	HLA-A*02:06	25.51
LVAEWFLAY	1505-1513	nsp3	HLA-A*01:01	26.28
NVFAFPFTI	5-13	ORF10	HLA-A*32:01	26.38
YINVFAFPF	3-11	ORF10	HLA-B*35:01	26.67
LIVAAIVFI	102-110	ORF7a	HLA-A*02:06	26.9
ITSGWTFGA	882-890	Surface glycoprotein	HLA-A*68:02	27.59
FLARGIVFM	184-192	nsp6	HLA-A*02:06	28.07
SELVIGAVI	136-144	Membrane glycoprotein	HLA-B*40:01	28.08
KLMGHFAWW	182-190	2′-O-ribosemethyl transferase	HLA-A*32:01	29.42
VLAWLYAAV	204-212	3C-like proteinase	HLA-A*02:06	29.5
AEWFLAYIL	1507-1515	nsp3	HLA-B*44:03	29.53
LSPRWYFYY	104-112	Nucleocapsid phosphoprotein	HLA-A*29:02	29.94
STNVTIATY	1455-1463	nsp3	HLA-A*30:02	30.16
WLMWLIINL	1545-1553	nsp3	HLA-A*02:06	31.04
KLFIRQEEV	85-93	ORF7a	HLA-A*02:01	31.81
LTNIFGTVY	433-441	nsp2	HLA-A*01:01	31.99
FLFLTWICL	26-34	Membrane glycoprotein	HLA-A*02:01	32.26
WEPEFYEAM	916-924	RNA-dependent RNA polymerase	HLA-B*18:01	32.4
VPWDTIANY	1315-1323	nsp3	HLA-B*53:01	33.04
KLINIIIWF	1407-1415	nsp3	HLA-A*32:01	33.93
VLWAHGFEL	184-192	3′-to-5′ exonuclease	HLA-A*02:06	34.55
HYVRITGLY	245-253	Helicase	HLA-A*29:02	35.21
VTWFHAIHV	62-70	Surface glycoprotein	HLA-A*68:02	35.91
LTNIFGTVY	433-441	nsp2	HLA-B*15:01	36.49
DYGARFYFY	599-607	nsp3	HLA-A*29:02	38.02
AANTVIWDY	80-88	Endo RNAse	HLA-A*30:02	39
HFYWFFSNY	389-397	nsp4	HLA-A*30:02	39.02
FSSEIIGYK	39-47	nsp4	HLA-A*11:01	39.17
VPFWITIAY	373-381	nsp4	HLA-B*18:01	39.28
WEPEFYEAM	916-924	RNA-dependent RNA polymerase	HLA-B*40:01	40.36
FMRFRRAFG	301-309	nsp4	HLA-B*08:01	41.75
FLRDGWEIV	461-469	nsp2	HLA-A*02:01	42.13
WPWYIWLGF	1212-1220	Surface glycoprotein	HLA-B*53:01	42.3
FELEDFIPM	263-271	Endo RNAse	HLA-B*40:02	42.48
HYVRITGLY	245-253	Helicase	HLA-A*30:02	43.01
SELVIGAVI	136-144	Membrane glycoprotein	HLA-B*40:02	43.47
QIGEYTFEK	194-202	Helicase	HLA-A*11:01	43.73
MPYFFTLLL	1351-1359	nsp3	HLA-B*39:01	44.17

Epitopes	Start-End	Protein	Predicted allotypes	Binding affinity (IC_{50})
ATAEAELAK	1796-1804	nsp3	HLA-A*11:01	46.33
TSAFVETVK	304-312	nsp2	HLA-A*11:01	46.54
VEHVTFFIY	225-233	ORF3a	HLA-B*44:03	47.43
FQVTIAEIL	7-15	ORF6	HLA-B*40:01	47.55
TLADAGFIK	827-835	Surface glycoprotein	HLA-A*11:01	48.34
LSPRWYFYY	104-112	Nucleocapsid phosphoprotein	HLA-A*01:01	48.64
NIALIWNVK	1890-1898	nsp3	HLA-A*68:01	49.44
MFLARGIVF	183-191	nsp6	HLA-A*23:01	50.53
FGDDTVIEV	7-15	nsp3	HLA-A*02:06	51.78
FLAFVVFLL	20-28	Envelope protein	HLA-A*02:06	51.99
MPYFFTLLL	1351-1359	nsp3	HLA-B*07:02	52.87
VFVLWAHGF	182-190	3′-to-5′ exonuclease	HLA-A*23:01	53.56
TLRVEAFEY	808-816	nsp3	HLA-A*29:02	55.46
CVDIPGIPK	39-47	3′-to-5′ exonuclease	HLA-A*11:01	55.47
AEWFLAYIL	1507-1515	nsp3	HLA-B*18:01	56.75
LFVAAIFYL	18-26	nsp4	HLA-A*23:01	57.15
KLMGHFAWW	182-190	2′-O-ribosemethyl transferase	HLA-B*58:01	57.81
IQYIDIGNY	71-79	ORF8	HLA-B*15:01	59.22
SHFAIGLAL	289-297	Helicase	HLA-B*38:01	60.43
FVAAIFYLI	19-27	nsp4	HLA-A*29:02	60.61
AYILFTRFF	1512-1520	nsp3	HLA-A*24:02	60.62
FELEDFIPM	263-271	Endo RNAse	HLA-B*35:01	60.81
TLADAGFIK	827-835	Surface glycoprotein	HLA-A*68:01	60.95
IMRLWLCWK	124-132	ORF3a	HLA-A*03:01	61.1
RMYIFFASF	1564-1572	nsp3	HLA-A*23:01	62.24
RMYIFFASF	1564-1572	nsp3	HLA-A*24:02	62.24
STNVTIATY	1455-1463	nsp3	HLA-B*15:01	62.85
IVAGGIVAI	284-292	nsp4	HLA-A*68:02	63.63
AYILFTRFF	1512-1520	nsp3	HLA-A*23:01	64.51
LTRNPAWRK	500-508	Helicase	HLA-A*03:01	67.1
SLREVRTIK	743-751	nsp3	HLA-A*03:01	67.66
KVSIWNLDY	23-31	ORF6	HLA-A*30:02	68.51
FWITIAYII	375-383	nsp4	HLA-A*23:01	68.87
VVYRAFDIY	30-38	RNA-dependent RNA polymerase	HLA-A*30:02	70.2
FLFVAAIFY	17-25	nsp4	HLA-B*35:01	71.25
LSPRWYFYY	104-112	Nucleocapsid phosphoprotein	HLA-A*30:02	74.89
REHEHEIAW	52-60	nsp2	HLA-B*44:03	75.78
RFRRAFGEY	303-311	nsp4	HLA-A*30:02	79.67
LMWLIINLV	1546-1554	nsp3	HLA-A*02:06	80.33
VFLFVAAIF	16-24	nsp4	HLA-A*24:02	80.73
IVAGGIVAI	284-292	nsp4	HLA-A*32:01	80.74
LLEDEFTPF	286-294	3C-like proteinase	HLA-B*15:01	81.14
SELVIGAVI	136-144	Membrane glycoprotein	HLA-B*44:03	82.78
YINVFAFPF	3-11	ORF10	HLA-B*15:01	83.83
IFLWLLWPV	52-60	Membrane glycoprotein	HLA-A*02:01	87.56

Epitopes	Start-End	Protein	Predicted allotypes	Binding affinity (IC_{50})
HVGEIPVAY	110-118	nsp1	HLA-A*29:02	89
KEGFFTYIC	146-154	2′-O-ribosemethyl transferase	HLA-B*40:02	95.5
FVAAIFYLI	19-27	nsp4	HLA-B*53:01	96.72
YRINWITGG	71-79	Membrane glycoprotein	HLA-B*27:05	98.22
FWITIAYII	375-383	nsp4	HLA-A*24:02	98.54
GEVITFDNL	730-738	nsp3	HLA-B*40:02	101.31
VFAFPFTIY	6-14	ORF10	HLA-A*30:02	101.96
HVTFFIYNK	227-235	ORF3a	HLA-A*30:01	104.38
VLLFLAFVV	17-25	Envelope protein	HLA-A*02:06	107.83
KRVDWTIEY	288-296	3′-to-5′ exonuclease	HLA-B*27:05	110.62
FLAFVVFLL	20-28	Envelope protein	HLA-A*68:02	111.56
FLNRFTTTL	219-227	3C-like proteinase	HLA-B*08:01	114.81
STKHFYWFF	386-394	nsp4	HLA-A*26:01	117.86
MLIIFWFSL	24-32	ORF7b	HLA-B*08:01	118.7
HVTFFIYNK	227-235	ORF3a	HLA-A*03:01	119.02
VPHVGEIPV	108-116	nsp1	HLA-B*07:02	119.11
GYAFEHIVY	229-237	Endo RNAse	HLA-A*29:02	120.67
VPFWITIAY	373-381	nsp4	HLA-B*53:01	120.82
AEWFLAYIL	1507-1515	nsp3	HLA-B*44:02	122.31
SQSIIAYTM	689-697	Surface glycoprotein	HLA-B*39:01	123.89
FELEDFIPM	263-271	Endo RNAse	HLA-B*39:01	125.67
LAYILFTRF	1511-1519	nsp3	HLA-B*35:01	132.38
FSYFAVHFI	1533-1541	nsp3	HLA-B*58:01	133.13
STKHFYWFF	386-394	nsp4	HLA-A*24:02	136.03
LAAECTIFK	152-160	nsp4	HLA-A*03:01	142.36
VFVLWAHGF	182-190	3′-to-5′ exonuclease	HLA-A*24:02	142.61
WEPEFYEAM	916-924	RNA-dependent RNA polymerase	HLA-B*40:02	144.78
VPFWITIAY	373-381	nsp4	HLA-A*29:02	148.55
MKIILFLAL	1-9	ORF7a	HLA-B*08:01	153.52
LTNIFGTVY	433-441	nsp2	HLA-A*29:02	155.95
IIFWFSLEL	26-34	ORF7b	HLA-A*32:01	157.05
FLNRFTTTL	219-227	3C-like proteinase	HLA-B*39:01	159
LAYILFTRF	1511-1519	nsp3	HLA-B*53:01	159.25
FLFLTWICL	26-34	Membrane glycoprotein	HLA-B*39:01	161.22
MPYFFTLLL	1351-1359	nsp3	HLA-B*51:01	162.35
STNVTIATY	1455-1463	nsp3	HLA-A*01:01	164.7
KLIFLWLLW	50-58	Membrane glycoprotein	HLA-B*57:01	170.58
LFLTWICLL	27-35	Membrane glycoprotein	HLA-A*23:01	171.35
RMYIFFASF	1564-1572	nsp3	HLA-B*08:01	172.56
FLFVAAIFY	17-25	nsp4	HLA-B*15:01	179.41
KLIFLWLLW	50-58	Membrane glycoprotein	HLA-A*23:01	182.96
MGYINVFAF	1-9	ORF10	HLA-A*23:01	183.8
ETTADIVVF	365-373	Helicase	HLA-A*26:01	187.4
PWYIWLGFI	1213-1221	Surface glycoprotein	HLA-A*23:01	187.82
FRLFARTRS	100-108	Membrane glycoprotein	HLA-B*27:05	188.55

Epitopes	Start-End	Protein	Predicted allotypes	Binding affinity (IC_{50})
LTNIFGTVY	433-441	nsp2	HLA-A*30:02	188.59
QHEETIYNL	81-89	RNA-dependent RNA polymerase	HLA-B*38:01	196.16
LAYILFTRF	1511-1519	nsp3	HLA-B*58:01	197.11
TLADAGFIK	827-835	Surface glycoprotein	HLA-A*03:01	198.58
LAYILFTRF	1511-1519	nsp3	HLA-B*15:01	198.91
QWSLFFFLY	30-38	nsp6	HLA-A*30:02	201.44
REHEHEIAW	52-60	nsp2	HLA-B*44:02	218.73
MRNAGIVGV	196-204	RNA-dependent RNA polymerase	HLA-B*39:01	219.69
AANTVIWDY	80-88	Endo RNAse	HLA-B*35:01	220.6
RFRRAFGEY	303-311	nsp4	HLA-A*29:02	221.2
MFLARGIVF	183-191	nsp6	HLA-A*24:02	223.67
HFYWFFSNY	389-397	nsp4	HLA-B*35:01	235.65
IFLWLLWPV	52-60	Membrane glycoprotein	HLA-A*23:01	236.36
STNVTIATY	1455-1463	nsp3	HLA-A*26:01	242.09
MRIFTIGTV	5-13	ORF3a	HLA-B*39:01	252.02
FQVTIAEIL	7-15	ORF6	HLA-B*48:01	258.85
KLMGHFAWW	182-190	2′-O-ribosemethyl transferase	HLA-B*57:01	268.05
ILFTRFFYV	1514-1522	nsp3	HLA-B*08:01	271.11
DLSPRWYFY	103-111	Nucleocapsid phosphoprotein	HLA-A*30:02	275.95
WNLVIGFLF	20-28	Membrane glycoprotein	HLA-A*23:01	277.26
FVAAIFYLI	19-27	nsp4	HLA-A*23:01	285.02
DSKEGFFTY	144-152	2′-O-ribosemethyl transferase	HLA-B*18:01	294.47
GHFAWWTAF	185-193	2′-O-ribosemethyl transferase	HLA-B*38:01	300.95
RMYIFFASF	1564-1572	nsp3	HLA-B*48:01	303.18
EHYVRITGL	244-252	Helicase	HLA-B*39:01	325.84
DGARRVWTL	134-142	nsp6	HLA-B*14:02	326.18
LIMLIIFWF	22-30	ORF7b	HLA-A*23:01	328.5
KVSIWNLDY	23-31	ORF6	HLA-A*01:01	333.91
RHVRAWIGF	81-89	3′-to-5′ exonuclease	HLA-A*24:02	362.97
MFLARGIVF	183-191	nsp6	HLA-B*35:01	365.15
LFTRFFYVL	1515-1523	nsp3	HLA-A*23:01	382.15
QQWGFTGNL	245-253	3′-to-5′ exonuclease	HLA-B*48:01	384.53
EHFIETISL	681-689	nsp3	HLA-B*38:01	397.15
AIVFITLCF	106-114	ORF7a	HLA-A*32:01	399.1
FVAAIFYLI	19-27	nsp4	HLA-A*32:01	417.26
KLIFLWLLW	50-58	Membrane glycoprotein	HLA-A*24:02	428.13
YINVFAFPF	3-11	ORF10	HLA-B*53:01	440.85
MPYFFTLLL	1351-1359	nsp3	HLA-B*08:01	454.87
DSKEGFFTY	144-152	2′-O-ribosemethyl transferase	HLA-A*26:01	458.91
LEGYAFEHI	227-235	Endo RNAse	HLA-B*40:01	463.52
RSFIEDLLF	815-823	Surface glycoprotein	HLA-A*32:01	465.9
EEEQEEDWL	154-162	nsp3	HLA-B*40:01	490.18
SELVIGAVI	136-144	Membrane glycoprotein	HLA-B*44:02	496.94
