# Supplementary material for: Immunogenic SARS-CoV-2 Epitopes: In Silico Study Towards Better Understanding of COVID-19 Disease—Paving the Way for Vaccine Development
Source: Vaccines (Basel). 2020 Jul 23;8(3):408. doi: 10.3390/vaccines8030408 (PMC7564651; doi:10.3390/vaccines8030408)
Supplement: Supplementary file 1 [file vaccines-08-00408-s001.zip › Table S7.pdf]

Table S7: Most potent SARS-CoV-2–derived MHC class I binding epitopes identified with NetCTL1.2 (combined score ≥ 2) prediction method.

Epitopes	Protein	Supertype	Combined score
CTDDNALAY	nsp9	A01	3.7840
FTSDYYQLY	ORF3a	A01	3.7470
PTDNYITTY	nsp3	A01	3.7170
LTDEMIAQY	Surface glycoprotein	A01	3.6616
DTDFVNEFY	RNA-dependent RNA polymerase	A01	3.6194
GTDLEGNFY	3C-like proteinase	A01	3.5954
ISDYDYYRY	RNA-dependent RNA polymerase	A01	3.2873
KSDGTGTIY	nsp9	A01	3.2109
AMDEFIERY	Endo RNAse	A01	3.1380
NSSTCMMCY	nsp3	A01	3.1372
LTNIFGTVY	nsp2	A01	3.1311
WTAGAAAYY	Surface glycoprotein	A01	3.1128
LTNDNTSRY	RNA-dependent RNA polymerase	A01	3.0815
TSNQVAVLY	Surface glycoprotein	A01	3.0758
FSAVGNICY	nsp4	A01	3.0377
TVNVLAWLY	3C-like proteinase	A01	2.9365
SSDNIALLV	Membrane glycoprotein	A01	2.9325
SSAKSASVY	nsp3	A01	2.8841
STNVTIATY	nsp3	A01	2.8727
FIDTKRGVY	nsp2	A01	2.8698
VVDKYFDCY	RNA-dependent RNA polymerase	A01	2.8284
LVAEWFLAY	nsp3	A01	2.7489
YTPSKLIEY	nsp4	A01	2.6909
KVSIWNLDY	ORF6	A01	2.6352
LTALRLCAY	Envelope	A01	2.6158
ATSRTLSYY	Membrane glycoprotein	A01	2.6146
ETISLAGSY	nsp3	A26	2.5793
CVADYSVLY	Surface glycoprotein	A01	2.5759
SSGDATTAY	RNA-dependent RNA polymerase	A01	2.5232
QSCTQHQPY	ORF8	A01	2.5149
QADVEWKFY	3′-to-5′ exonuclease	A01	2.4691
AANTVIWDY	Endo RNAse	A01	2.4255
TTTIKPVTY	nsp3	A01	2.4247
CASEYTGNY	nsp3	A01	2.4129
LMNVLTLVY	nsp6	A01	2.4071
SHVVAFNTL	nsp4	B39	2.4037
TVAYFNMVY	nsp6	A01	2.4032
KTSVDCTMY	Surface glycoprotein	A01	2.3795
LTGHMLDMY	RNA-dependent RNA polymerase	A01	2.3757
STECSNLLL	Surface glycoprotein	A01	2.3492
LSPRWYFYY	Nucleocapsid phosphoprotein	A01	2.3408
QHEETIYNL	RNA-dependent RNA polymerase	B39	2.3379
FKEGSSVEL	RNA-dependent RNA polymerase	B39	2.3210
FVFKNIDGY	Surface glycoprotein	A26	2.2795

Epitopes	Protein	Supertype	Combined score
YHPNCVNCL	RNA-dependent RNA polymerase	B39	2.2748
DVTDVTQLY	Helicase	A26	2.2677
YTERSEKSY	nsp2	A01	2.2636
FLTENLLLY	nsp3	A01	2.2528
ASHMYCSFY	nsp3	A01	2.2414
VVDYGARFY	nsp3	A01	2.2399
GTFTCASEY	nsp3	A01	2.2118
NQDLNGNWY	RNA-dependent RNA polymerase	A01	2.1915
FVVEVVDKY	RNA-dependent RNA polymerase	A26	2.1853
QTFSVLACY	3C-like proteinase	A26	2.1826
NPAWRKAVF	Helicase	B08	2.1824
HTDLMAAYV	nsp3	A01	2.1725
SHFAIGLAL	Helicase	B39	2.1687
EHFIETISL	nsp3	B39	2.1674
TLQCIMLVY	nsp6	A01	2.1660
DTVIEVQGY	nsp3	A26	2.1628
QHMVVKAAL	3′-to-5′ exonuclease	B39	2.1615
YLKLRSDVL	nsp4	B08	2.1523
YLRKHFSMM	RNA-dependent RNA polymerase	B08	2.1491
EIVDTVSAL	Helicase	A26	2.1488
FYGGWHNML	RNA-dependent RNA polymerase	B39	2.1432
VTDVTQLYL	Helicase	A01	2.1239
ETKDVVECL	nsp3	A26	2.0926
SCNNYMLTY	nsp3	A01	2.0891
TITQMNLKY	RNA-dependent RNA polymerase	A01	2.0878
MSNLGMPSY	nsp3	A01	2.0858
FTSDYYQLY	ORF3a	A26	2.0795
TILDGISQY	nsp2	A26	2.0747
YTPSKLIEY	nsp4	A26	2.0734
YADVFHLYL	RNA-dependent RNA polymerase	B39	2.0725
FNEKTHVQL	nsp1	B39	2.0723
YIKWDLLKY	RNA-dependent RNA polymerase	A26	2.0667
YYTSNPTTF	nsp3	A24	2.0661
AHAEETRKL	nsp3	B39	2.0425
SSPDAVTAY	nsp3	A01	2.0391
AEWFLAYIL	nsp3	B44	2.0360
YFMRFRRAF	nsp4	B08	2.0284
TQYNRYLAL	nsp4	B39	2.0256
DVFHLYLQY	RNA-dependent RNA polymerase	A26	2.0054
WTAGAAAYY	Surface glycoprotein	A26	2.0048
GHSMQNCVL	3C-like proteinase	B39	2.0004
