# Supplementary material for: Immunogenic SARS-CoV-2 Epitopes: In Silico Study Towards Better Understanding of COVID-19 Disease—Paving the Way for Vaccine Development
Source: Vaccines (Basel). 2020 Jul 23;8(3):408. doi: 10.3390/vaccines8030408 (PMC7564651; doi:10.3390/vaccines8030408)
Supplement: Supplementary file 1 [file vaccines-08-00408-s001.zip › Table S8.pdf]

Table S8: SARS-CoV-2–derived MHC class I binding epitopes that are identical to the most potent, experimentally known epitopes (MHC ligand assay data from the IEDB database) of SARS-CoV strains that activate cytotoxic T-cells. Predicted half-lives of the epitope–MHC-I complexes, GRAVY scores and mutations in the epitopes are shown.

SARS-CoV-2 epitopes	Start-End	SARS-CoV-2 Protein	Mutation in epitope	GRAVY score	Allotypes	Half-life (in hours)	Predicted IC_{50} (nM)	Experimental IC_{50} (nM)/ IEDB reference	Matching Epitope IEDB ID	Matching epitope Start-End	SARS Proteins	UniProt ID	SARS Species
AANTVIWDY	80-88	Endo RNAse	A81V<br>Y88C	0.267	A*30:02<br>B*15:25<br>B*35:01	0.90<br>0.58<br>1.51	39<br>-<br>220.60	-<br>-<br>-	340	6509-6517	Polyprotein 1ab	P0C6X7	SARS-CoV Tor2
CVDIPGIPK	39-47	3′-to-5′ exonuclease	P46L	0.522	A*11:01	1.40	55.47	19.09 / 1000425	7258	5941-5949	Polyprotein 1ab	P0C6X7	SARS-CoV Tor2
DSKEGFFTY	144-152	2′-O-ribosemethyl transferase	Y152H	-0.944	A*25:01<br>A*26:01<br>B*15:25<br>B*18:01	0.53<br>0.50<br>0.33<br>0.30	935.74<br>458.91<br>-<br>294.47	-<br>993 / 1000425<br>-<br>-	10151	6919-6927	Polyprotein 1ab	P0C6X7	SARS-CoV Tor2
EHYVRITGL	244-252	Helicase	No	-0.122	B*39:01<br>B*52:01	0.47<br>0.21	325.84<br>-	-<br>-	12380	5545-5553	Polyprotein 1ab	P0C6X7	SARS-related-CoV
FIAGLIAIV	1220-1228	Surface glycoprotein	No	3.56	A*02:01<br>A*02:06<br>A*25:01<br>A*26:01<br>A*68:02	5.11<br>5.42<br>0.27<br>0.36<br>0.63	10.29<br>11.13<br>5479.67<br>802.95<br>8.32	1.48 / 1000425<br>2.8 / 1000425<br>-<br>-<br>0.54 / 1000425	16156	1202-1210	Surface glycoprotein	P59594	SARS-CoV Tor2
FLAFVVFLL	20-28	Envelope protein	No	3.333	A*02:01<br>A*02:06<br>A*68:02	11<br>5.48<br>0.44	5.26<br>51.99<br>111.56	0.23 / 1000425<br>2.57 / 1000425<br>391 / 1000425	16501	20-28	Envelope protein	P59637	SARS-CoV Tor2
FLNRFTTTL	219-227	3C-like proteinase	L220F	0.344	A*02:01<br>B*08:01<br>B*15:02<br>B*15:25<br>B*39:01	13.57<br>0.26<br>1.55<br>1.73<br>0.43	9.14<br>114.81<br>1407.69<br>-<br>159	8 / 1000425<br>-<br>-<br>-<br>-	16786	3459-3467	Polyprotein 1a	P0C6U8	SARS-CoV
GSVGFNIDY	146-154	3C-like proteinase	No	0.178	B*15:25	0.62	-	-	22560	3386-3394	Polyprotein 1ab	P0C6X7	SARS-CoV Tor2
GYAFEHIVY	229-237	Endo RNAse	No	0.400	A*29:02	0.42	120.67	476 / 1000425	23303	6658-6666	Polyprotein 1ab	P0C6X7	SARS-CoV Tor2
HYVRITGLY	245-253	Helicase	No	0.122	A*26:01<br>A*29:02<br>A*30:02	0.32<br>1.86<br>1.35	1091.74<br>35.21<br>43.01	149 / 1000425<br>111 / 1000425<br>-	25208	5546-5554	Polyprotein 1ab	P0C6X7	SARS-CoV Tor2
KLFIRQEEV	85-93	ORF7a	No	-0.400	A*02:01	3.17	31.81	400.25 / 1000945	31846	85-93	Protein7a	P59635	SARS-related-CoV
LLDDFVEII	298-306	Endo RNAse	No	1.456	A*02:01<br>B*13:02<br>B*52:01	5.82<br>0.45<br>0.55	23.67<br>-<br>-	51 / 1000425<br>-<br>-	37144	6727-6735	Polyprotein 1ab	P0C6X7	SARS-CoV Tor2
LLSAGIFGA	330-338	nsp3	I335V	1.878	A*02:01<br>A*02:06	2.90<br>1.45	10.09<br>14.54	8.1 / 1000425<br>24.6 / 1000425	37766	1126-1134	Polyprotein 1ab	P0C6X7	SARS-related-CoV
LSPRWYFYY	104-112	Nucleocapsid phosphoprotein	No	-0.567	A*01:01<br>A*29:02<br>A*30:02	1.34<br>1.48<br>0.84	48.64<br>29.94<br>74.89	38.1 / 1000425<br>2.58 / 1000425<br>66.5 / 1000425	39576	105-113	Nucleoprotein	P59595	SARS-CoV Tor2
LTRNPAWRK	500-508	Helicase	P504L	-1.556	A*03:01<br>A*30:01<br>A*74:01	0.90<br>1.40<br>0.68	67.10<br>12.41<br>-	222 / 1000425<br>-<br>-	40154	5801-5809	Polyprotein 1ab	P0C6X7	SARS-CoV Tor2
MLIIFWFSL	24-32	ORF7b	F28Y<br>S31L	2.489	A*02:01<br>A*02:06<br>A*32:01<br>B*08:01	2.12<br>1.68<br>0.31<br>0.19	13.26<br>25.51<br>15.86<br>118.70	1160 / 1000425<br>490 / 1000425<br>-<br>-	41962	24-32	Protein non-structural 7b	Q7TFA1	SARS-CoV Tor2
NGDVVAIDY	1146-1154	nsp3	No	0.278	A*01:01	0.31	731.96	1030 / 1000425	43972	1941-1949	Polyprotein 1ab	P0C6X7	SARS-CoV Tor2
NLWNTFTRL	518-526	3′-to-5′ exonuclease	T524I	-0.378	B*13:01	0.32	-	-	44927	6420-6428	Polyprotein 1ab	P0C6X7	SARS-related-CoV
NVLAWLYAA	203-211	3C-like proteinase	No	1.278	A*02:06	1.85	8.31	1.58 / 1000425	46414	3443-3451	Polyprotein 1ab	P0C6X7	SARS-related-CoV
QIGEYTFEK	194-202	Helicase	K202R	-1.056	A*11:01<br>A*74:01	3.05<br>0.37	43.73<br>-	9.93 / 1000425<br>-	51068	5495-5503	Polyprotein 1ab	P0C6X7	SARS-CoV Tor2
RMYIFFASF	1564-1572	nsp3	No	1.111	A*23:01<br>A*24:02<br>A*32:01<br>A*74:01<br>B*08:01<br>B*15:01<br>B*15:25<br>B*46:01<br>B*48:01<br>B*52:01	2.05<br>0.98<br>3.50<br>0.34<br>0.20<br>6.76<br>1.39<br>0.43<br>0.61<br>0.40	62.24<br>62.24<br>4<br>-<br>172.56<br>5.53<br>-<br>3225.58<br>303.18<br>-	75.3 / 1000425<br>106 / 1000425<br>-<br>-<br>-<br>-<br>-<br>-<br>-<br>-	54960	2359-2367	Polyprotein 1ab	P0C6X7	SARS-CoV Tor2
SELVIGAVI	136-144	Membrane glycoprotein	No	2.033	B*13:01<br>B*13:02<br>B*37:01<br>B*40:01<br>B*40:02<br>B*44:02<br>B*44:03<br>B*49:01<br>B*50:01<br>B*52:01	0.63<br>0.44<br>0.63<br>1.07<br>2.72<br>0.77<br>1.08<br>0.70<br>0.30<br>0.63	-<br>-<br>-<br>28.08<br>43.47<br>496.94<br>82.78<br>-<br>-<br>-	-<br>-<br>-<br>88.9 / 1000425<br>55.9 / 1000425<br>605 / 1000425<br>426 / 1000425<br>-<br>-<br>-	57544	135-143	Membrane protein	P59596	SARS-CoV Tor2
SPRWYFYYL	105-113	Nucleocapsid phosphoprotein	No	-0.567	B*07:02<br>B*08:01<br>B*35:03<br>B*55:01<br>B*56:01	1.77<br>0.31<br>0.50<br>0.25<br>0.34	6.32<br>13.77<br>4757.16<br>-<br>-	2.86 / 1000425<br>-<br>-<br>-<br>-	60242	106-114	Nucleoprotein	P59595	SARS-CoV Tor2
SRYWEPEFY	913-921	RNA-dependent RNA polymerase	No	-1.622	B*15:25<br>B*27:02	0.35<br>1.39	-<br>-	-<br>-	60868	5282-5290	Polyprotein 1ab	P0C6X7	SARS-related-CoV
VLAWLYAAV	204-212	3C-like proteinase	No	2.133	A*02:01<br>A*02:06	8.13<br>4.42	13.40<br>29.50	0.435 / 1000425<br>8.79 / 1000425	69392	3444-3452	Polyprotein 1a	P0C6U8	SARS-CoV
VLLFLAFVV	17-25	Envelope protein	No	3.489	A*02:01<br>A*02:06	4.13<br>1.90	21.72<br>107.83	5.62 / 1000425<br>12.6 / 1000425	69591	17-25	Envelope protein	P59637	SARS-related-CoV
VLWAHGFEL	184-192	3′-to-5′ exonuclease	No	0.933	A*02:01<br>A*02:06<br>B*13:01<br>B*13:02<br>B*15:25<br>B*52:01	6.51<br>2.26<br>0.70<br>0.33<br>0.65<br>0.31	5.78<br>34.55<br>-<br>-<br>-<br>-	0.40 / 1000425<br>20.3 / 1000425<br>-<br>-<br>-<br>-	69850	6086-6094	Polyprotein 1ab	P0C6X7	SARS-related-CoV
VVYRAFDIY	30-38	RNA-dependent RNA polymerase	No	0.767	A*30:02<br>B*15:25	0.79<br>0.88	70.20<br>-	-<br>-	71916	4399-4407	Polyprotein 1ab	P0C6X7	SARS-CoV Tor2
