# Supplementary material for: Immunogenic SARS-CoV-2 Epitopes: In Silico Study Towards Better Understanding of COVID-19 Disease—Paving the Way for Vaccine Development
Source: Vaccines (Basel). 2020 Jul 23;8(3):408. doi: 10.3390/vaccines8030408 (PMC7564651; doi:10.3390/vaccines8030408)
Supplement: Supplementary file 1 [file vaccines-08-00408-s001.zip › Table S9.pdf]

Table S9: Most frequently recorded intermolecular hydrogen bond interactions during the replicate 100 ns simulations.

A. HLA-A*02:01-epitope interactions

**HLA-A*02:01**	**Epitope**	**Frequency** **(Simulation1, Simulation2, Simulation3)**
THR_143@OG1	VAL_1228@O	98%, 90%, 40%
TRP_147@NE1	ILE_1227@O	97%, 97% 71%
Arg_97@NH2	Leu_1224@O	18%, 97%, 67%
TYR_99@OH	ALA_1222@N	62%, 50%, 24%
ASP_77@OD1	VAL_1228@N	47%, 78%, 99%

B. TCR-epitope interactions

**TCR**	**Epitope**	**Frequency** **(Simulation1, Simulation2, Simulation3)**
TRP_99@NE1 (β chain)	ILE_1225@O	63%, 73%, 13%
GLN_101@NE2 (α chain)	ILE_1225@O	10%, 19%, 25%

C. HLA-A*02:01-TCR interactions

**HLA-A*02:01**	**TCR**	**Frequency** **(Simulation1, Simulation2, Simulation3)**
THR_80@OG1	ASN_51@ND2 (β chain)	24.6%, 13%, 16%
ARG_17@NH2	GLU_53@OE2 (β chain)	21%, 13%, 10%
ARG_17@NH2	GLU_53@OE1 (β chain)	20.5%, 12%, 11%
ARG_17@NH1	GLU_53@OE1 (β chain)	18%, 12%, 10%
LYS_146@NZ	GLU_30@OE1 (β chain)	15.4%, 10%, 21%
GLN_155@OE1	ASN_32@ND2 (α chain)	15.2%, 13%, 15.3%
